# Molecular Dynamics of Janus Nanodimers Dispersed in Lamellar Phases of a Block Copolymer

**DOI:** 10.3390/polym13091524

**Published:** 2021-05-09

**Authors:** J. Javier Burgos-Mármol, Alessandro Patti

**Affiliations:** 1 Institute of Systems, Molecular & Integrative Biology, University of Liverpool, Crown St., Liverpool L69 7ZB, UK; J.J.Burgos-Marmol@liverpool.ac.uk; 2 Department of Chemical Engineering and Analytical Science, The University of Manchester, The Mill. Sackville Street, Manchester M13 9PL, UK

**Keywords:** polymer nanocomposites, molecular dynamics, nanoparticles, janus, copolymer, lamellar, coarse-grained, nematic, orientational order, diffusion

## Abstract

We investigate structural and dynamical properties of Janus nanodimers (NDs) dispersed in lamellar phases of a diblock copolymer. By performing molecular dynamics simulations, we show that an accurate tuning of the interactions between NDs and copolymer blocks can lead to a close control of NDs’ space distribution and orientation. In particular, NDs are preferentially found within the lamellae if enthalpy-driven forces offset their entropic counterpart. By contrast, when enthalpy-driven forces are not significant, the distribution of NDs, preferentially observed within the inter-lamellar spacing, is mostly driven by excluded-volume effects. Not only does the degree of affinity between host and guest species drive the NDs’ distribution in the polymer matrix, but it also determines their space orientation. In turn, these key structural properties influence the long-time dynamics and the ability of NDs to diffuse through the polymer matrix.

## 1. Introduction

Polymer nanocomposites (PNCs) are hybrid materials comprising a host polymer matrix embodying guest nanoparticles (NPs). They couple many of the advantageous characteristics of polymers, such as flexibility, processability and ductility, to enhanced thermal and mechanical stability as well as optical, electrical, magnetic and other intriguing NPs’ properties. Incorporating nano-sized rather than micron-sized fillers poses a comparatively relevant difference in terms of available polymer/NP interface, where the interactions between the two species are able to enhance the material performance by triggering new properties [1]. In particular, NPs display a surface area-to-volume ratio that is orders of magnitude larger than that provided by similarly shaped micrometric particles, with the tremendous potential of generating new properties at a relatively low loading [2]. This is not of secondary importance as the resulting material’s weight can be kept very close to that of the pristine polymer, with evident benefits in industrial applications requiring light materials, such as low CO${}_{2}$ emission vehicles where metal components, heavier and prone to corrosion, are gradually being substituted by PNCs [3]. PNCs are also being investigated for applications in food packaging to offer high barrier to diffusion of atmospheric gases [4], enhanced thermal properties for cook-in-bag or microwavable packages [5] and even to detect undesired volatile molecules that would flag poor food integrity or spoilage as a more precise alternative to uncertain sell-by dates [6]. Another intriguing cutting-edge research field where PNCs are showing a promising potential is that of organic solar cells. In this context, PNCs can provide, e.g., enhanced protection to UV light, flexibility, transparency and more contained costs of production and maintenance as compared to conventional silicon cells [3,7,8].

Despite these fascinating technological opportunities, there are still pressing issues hampering the implementation of PNCs in large-scale applications and an accurate control of their impressive potential [9]. These issues include the difficulty of uniformly dispersing NPs in the polymer matrix and avoiding the formation of separate clusters [10,11,12,13]. Not only would these clusters of NPs leave the polymer’s properties practically unchanged, but they could also trigger the occurrence of structural defects. Therefore, significant effort has been devoted to enhance the NP miscibility by chemically modifying their surface with polymer grafts or ligand molecules [14,15,16,17,18]. This sort of problems is also being addressed by theoretical methods. These include field-theoretic approaches, such as the self-consistent field theory (SCFT) and the single-chain mean-field theory, currently mostly applied to pure block copolymers [19,20,21]. The phase behaviour of PNCs can be studied by combining SCFT with density functional theory [22,23], but their accuracy in the description of the orientation of anisotropic nanoparticles is still quite limited [24]. Alternatively, hybrid particle-field approaches can be considered, where particles are explicitly modelled, but the polymer chains are treated in a mean-field fashion [25,26,27]. Equally relevant in case of anisotropic NPs is controlling their orientation in the polymer matrix, for instance to enhance barrier properties against gas permeability [4] or to modulate their optical response and induce fluorescence anisotropy [28,29]. Due to their amorphous or semicrystalline morphology, most polymers offer a relatively low barrier to molecular permeability and diffusion, which two-dimensional NPs, by developing a rather tortuous permeation network, could in principle improve [30]. Nevertheless, the simple incorporation of well-dispersed nanoplatelets might have a very limited benefit if not accompanied by a close control on their orientation [31,32]. To this end, by orienting 2 wt% graphene oxide nanoplatelets in compressed polystyrene film coatings, Wan and coworkers very recently observed a 96% reduction in oxygen permeation as compared to the pristine polymer [33]. Similar to their oblate counterparts, prolate NPs also disclose a wide spectrum of fascinating applications. For instance, noble metal nanorods are excellent NPs to amplify local electric fields, while semiconducting nanorods enhance the efficiency of polymer-based photovoltaic devices by increasing electron mobility and hence electrical conductivity [34]. The latter displays a complex dependence on the degree of nanorods’ alignment. More specifically, it increases by slightly orienting the nanorods, but, when these are highly aligned, it shows a drastic decrease, especially at low anisotropies and volume fractions [35]. There are different options to align nanorods or similar one-dimensional NPs, such as nanowires and nanotubes [36,37,38,39], but none of them, for the time being, seem to exhibit a notably more accurate control on particle orientation than the others.

Although the design and production of PNCs incorporating positionally and orientationally ordered anisotropic NPs implies severe experimental challenges, the general consensus is that spatial organisation is instrumental to fully exploit their technological potential [40,41,42,43,44,45,46]. Realising that spatial organisation can be tailored by simultaneously controlling enthalpy and entropy-driven forces fuelled the interest in PNCs consisting of block copolymers (BCPs) and functional NPs [47]. The reason these mixtures are especially suitable to tune enthalpic and entropic contributions is because they present microphase separated domains promoting favourable NP/BCP thermodynamic and geometric interactions that can accurately direct NPs’ position and orientation [32,48,49]. Most of the research work on these systems has been limited to BCPs incorporating spherical NPs or, to a much lesser extent, rod-like [46,50] and disk-like [32,51] anisotropic NPs. Nevertheless, studies on non-spherical NPs that exhibit two chemically distinct compartments and are therefore anisotropic in both shape and interactions are still at a stage where theory and simulation are paving the path for experiments [26,44,52,53,54]. Such a double anisotropy provides additional degrees of freedom to more precisely control the interplay between enthalpy and entropy by tuning NP size, shape and surface chemistry, in relation to the architecture of the BCP employed and its potential ability of self-assembling into periodically ordered mesophases. By preferentially orienting at the interface between the two copolymer domains, these intriguing NPs, generally referred to as Janus NPs after the double-faced Roman god, provide an opportunity to better control the functionalisation of the BCP for an ample spectrum of industrial applications.

In this work, we perform molecular dynamics (MD) simulations to investigate the structural and dynamical properties of Janus and homogeneous nanodimers (NDs) embedded in lamellar phases of a diblock copolymer (di-BCP) melt. To this end, rather than an atomistic model that would be too computationally demanding to explore the relevant time and length scales of PNCs, we opt for a coarse-grained representation of the polymer chains, a strategy that in the past allowed us to study the behaviour of BCPs in solutions [55,56,57,58,59,60,61] and melts [62,63,64]. Coarse-grained models portray groups of atoms as single interaction sites, reducing the number of degrees of freedom and thus allowing the study of longer time and length scales. Even though this reductionist model lacks full chemical detail, it still proves to be an efficient method to study properties at the mesoscale such as self-assembly [65,66], phase behaviour [67], structure [68], dynamics [69] and viscoelastic and thermal properties [70,71]. Our interest is understanding how the interactions between the polymer chain and the NDs impact distribution and orientation of the particles in the lamellae and how this ordering (or lack of) influences their mobility at long-time scales.

## 2. Model and Simulation Methodology

In this section, we discuss the model employed to mimic the behaviour of a di-BCP hosting NDs, the computational details of our MD simulations and the characterisation techniques to determine structural and dynamical properties of the model PNC.

### 2.1. Molecular Model

**Figure 1:**
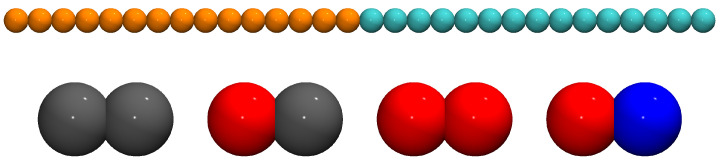
Model di-BCP chain with h-block beads in orange and t-block beads in cyan (**top**); and model nanodimers with neutral (grey), head-like (red) and tail-like (blue) beads (**bottom**).

Coarse-grained (CG) models of di-BCP chains and NDs have been applied to generate ordered lamellar phases with specific NP content and chain-dimer interaction, as detailed in Section 2.2. By disregarding computationally expensive chemical details, CG models allow to simulate the mesoscale behaviour of materials, which fully evolves over considerable length and time scales. While of fundamental importance at the molecular scale, these degrees of freedom are less and less relevant at increasing time and length scales to determine, for instance, chain conformations and structural relaxation [62,72,73,74,75]. To coarse-grain a polymer chain, a small portion of the macromolecule is reduced to a single interaction site, here referred to as bead, whose interactions average the contributions of the atoms it comprises. More specifically, our model copolymer chain consists of l=30 beads separated into a head block (h) and a tail block (t) of 15 beads each. This value was chosen to obtain lamellar layers thicker than the size of the NDs and systems with enough polymer chains to minimise finite-size effects. At the same time, it is not too large to hamper the study of the long-time diffusive regime within our simulation time. Each chain bead has a diameter $\sigma_{h}=\sigma_{t}=\sigma$ and a mass $m_{h}=m_{t}=M$, which define, respectively, the system’s unit length and unit mass in Lennard–Jones (LJ) units. Additionally, NDs consist of two beads of diameter $\sigma_{H}=\sigma_{T}=\sigma_{0}=3\;\sigma$ and mass $m_{H}=m_{T}=m_{0}=19.59\;M$, which are bonded to each other with a small overlap (see Section S1 of the Supplementary Materials for a detailed calculation of the NDs’ mass). Depending on their chemical affinity to the two chain blocks, ND beads can be head-like (H), tail-like (T) or neutral (0). A schematic representation of a copolymer chain and the full set of the NDs studied in this work is shown in Figure 1.

The force-field defining the interactions between the different components of our systems is a combination of the models proposed by Kremer and Grest for polymer chains [76] and Smith et al. for NPs [77]. These models have been applied in the past to investigate structural and dynamical properties across several length and time scales [72,73,78,79]. In particular, non-bonded interactions are described by a shifted Weeks–Chandler–Andersen (WCA) potential [80] that reads:

(1) $$U_{\alpha \beta }\left(r\right)=\left\{4\epsilon_{\alpha \beta }\left[{\left(\frac{\sigma }{r-\Delta_{\alpha \beta }}\right)}^{12}-{\left(\frac{\sigma }{r-\Delta_{\alpha \beta }}\right)}^{6}\right]+\epsilon_{\alpha \beta }\right\}\times H\left[-(r-\Delta_{\alpha \beta }-r_{c})\right]$$

where $\epsilon_{\alpha \beta }$ is the intensity of the force between beads α and β, with α,β∈{h,t,H,T,0}; $\Delta_{\alpha \beta }$ is a shifting parameter, H[x] is the Heaviside step distribution; and $r_{c}=2^{1/6}\;\sigma$ is the cutoff distance. The limits of this potential are $U_{\alpha \beta }\left(r-\Delta_{\alpha \beta }⩽0\right)=\infty$ and $U_{\alpha \beta }\left(r-\Delta_{\alpha \beta }⩾r_{c}\right)=0$. The parameter $\Delta_{\alpha \beta }\neq 0$ effectively provides the beads with a hard core and a soft shell [64]. The values of $\Delta_{\alpha \beta }$ and $\epsilon_{\alpha \beta }$ have been chosen to favour the formation of a lamellar phase while hampering NP aggregation [81,82]. The cutoff distance is the typical one of the WCA potential, which drops the attractive range of the LJ function, thus turning it into a purely repulsive potential. The complete parameterisation of this potential is provided in Table 1.

To describe the interaction between two contiguous chain beads as well as between two beads of the same dimer, we applied the Finitely Extensible Non-linear Elastic (FENE) potential combined with a WCA potential, which reads [76]:

(2) $$\begin{array}{cc} V_{\mathrm{stretching}}\left(r\right) & =-\frac{1}{2}K_{\alpha \beta }R_{0,\alpha \beta }^{2}\ln\left[1-{\left(\frac{r}{R_{0,\alpha \beta }}\right)}^{2}\right]+\\ \; & +\left\{4\epsilon \left[{\left(\frac{\sigma_{\alpha \beta }}{r}\right)}^{12}-{\left(\frac{\sigma_{\alpha \beta }}{r}\right)}^{6}\right]+\epsilon \right\}\times H\left[-(r-r_{c})\right] \end{array}$$

where $K_{\alpha \beta }$ and $R_{0,\alpha \beta }$ are the intensity and range of the interaction, respectively; $\sigma_{\alpha \beta }=\left(\sigma_{\alpha }+\sigma_{\beta }\right)/2$; and ϵ is the system’s unit energy. Polymer chain bonds, i.e., h-h, h-t and t-t bonds, are parameterised as proposed by Kremer and Grest [76]. However, the force-field parameters used for the beads forming Janus and homogeneous NDs have been derived specifically for this work and are set in such a way to allow a partial overlap between them and a mutual distance $d_{\mathrm{ND}}\approx 2.5\;\sigma$. The complete set of bonded interaction parameters is reported in Table 1.

### 2.2. Simulation Methodology

We studied the structural and dynamical properties of the systems listed in Table 2, which also include a reference system (PNC${}_{0}$) containing polymer only. All systems consist of lamellar phases incorporating 12,696 diblock copolymer chains and 96, 510 or 1080 NDs, corresponding to 1 wt%, 5 wt% or 10 wt%, respectively. These mass fractions are those usually employed in experimental PNCs. Below 1 wt%, it is difficult to see tangible modifications of the macroscopic material properties, and above 10 wt% the long-range ordering might be destroyed [83]. All systems were equilibrated and characterised by performing MD simulations on the Large-scale Atomic/Molecular Massively Parallel Simulator (LAMMPS) [84,85]. All MD simulations were parallel-run on 4 Intel Skylake nodes (160 CPUs) [86].

The time-step for all the simulations was fixed at δt=0.001τ, with $\tau =\sigma M^{1/2}\epsilon^{-1/2}$ the system’s unit time. Equilibration and production runs were performed in the isothermal-isobaric (NPT) ensemble, i.e., at constant temperature $T=\epsilon /k_{B}$, with $k_{B}$ the Boltzmann constant, constant pressure $P=11.3\;\epsilon \sigma^{-3}$ and constant number of chains and dimers. To this end, a Nosé–Hoover barostat and thermostat were used [87,88].

**Figure 2:**
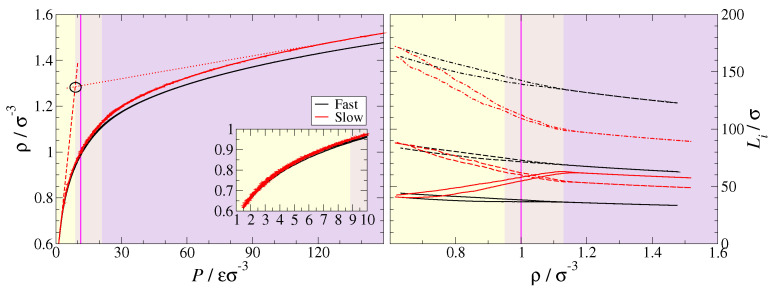
Isothermal curves at $T=1\;\epsilon /k_{B}$ and pressure rates $\delta P/\delta t=1.5\cdot 10^{-2}\;\epsilon \sigma^{-3}\tau^{-1}$ (black) and $\delta P/\delta t=3.0\cdot 10^{-3}\;\epsilon \sigma^{-3}\tau^{-1}$ (red) obtained by expanding and then compressing between $P_{1}=1.5\;\epsilon \sigma^{-3}$ and $P_{2}=150\;\epsilon \sigma^{-3}$. The continuous vertical line marks our system’s pressure $P=11.3\;\epsilon \sigma^{-3}$ and density $\rho =1.0\;\sigma^{-3}$ (magenta). (**Left**) Pressure vs. density plane. It contains the isotherms (continues lines) and linear regressions of the slow-rate curve at each end (dashed and dotted red lines). Their intersection ($P=8.82\;\epsilon \sigma^{-3},\rho =0.95\;\sigma^{-3}$) distinguishes isotropic (light yellow area) from lamellar (yellow-purple mixed-coloured and purple areas) phases. The inset shows the same results in the low-density region. (**Right**) Box dimensions vs. density plane. It displays evolution of $L_{x}$ (dashed lines), $L_{y}$ (dashed-dotted lines) and $L_{z}$ (continuous lines) at the two pressure rates. An abrupt change in $\delta L_{i}/\delta \rho$ is observed at $\rho =1.13\;\sigma^{-3}$.

Systems were considered to be at equilibrium when the total energy *E* and the volume $V_{\mathrm{box}}$ achieved a steady value within reasonable statistical fluctuations, which involved runs of $2–3\cdot 10^{8}\;\delta t$. We also monitored the fluctuations of the box length perpendicular to lamellar-planes $L_{z}$ and made sure these were also very small at equilibrium. The pressure was set to a value that produced a number density of $\rho =1.0\;\sigma^{-3}$ in the pristine polymer, right in the faster-dynamics section of the lamellar phase (see equations of state in Figure 2). We note that energy minimisation was generally observed right after reaching the target temperature and pressure, suggesting that, for most of the equilibration run, the structural relaxation of the system was mostly due to entropic forces. A fully detailed description of the equilibration process is provided in Section S2, including Figures S1 and S2. [89,90,91,92] Production runs of $t=10^{8}\;\delta t$ were then performed to generate trajectories at both linear and logarithmic time intervals that would be used for subsequent data analysis.

### 2.3. Data Analysis

To characterise the morphology of our model PNC, we computed the density profiles perpendicularly to the lamellae as well as the distribution and orientation of dimers and chains. The homemade software, written in FORTRAN 90, is available online to the interested reader [93]. In particular, the density profiles provide the number density of component α in rectangular bins parallel to the lamellae:

(3) $$\rho_{\alpha }\left(z_{i}\pm \delta z\right)=\frac{{\langle n_{\alpha }\left(z_{i}\pm \delta z\right)\rangle }_{i}}{V_{\mathrm{bin}}}$$

where δz is half the width of bin *i*, $\rho_{\alpha }$ is the local density of component α, $n_{\alpha }$ is the number of beads of component α in *i*, $V_{\mathrm{bin}}=2L_{x}L_{y}\delta z$ is the volume of bin *i* and ${\langle ...\rangle }_{i}$ indicates ensemble average. The orientational distribution of dimers is especially useful to understand how the polymer–dimer interactions affect the dimer orientation in space, i.e., the orientation of the vector connecting the centres of mass of the dimer beads, ${\vec{d}}_{\mathrm{ND}}$. To estimate this distribution, we determined the orientation by measuring the polar angle $\theta \in [-90^{\circ },90^{\circ }]$, its absolute value and the azimuthal angle $\phi \in [-180^{\circ },180^{\circ })$. More specifically:

(4) $$\langle \theta \rangle =\langle \arccos\left({\hat{d}}_{\mathrm{ND}}\cdot {\hat{e}}_{z}\right)-90^{\circ }\rangle$$

(5) $$\langle |\theta |\rangle =\langle |\arccos\left({\hat{d}}_{\mathrm{ND}}\cdot {\hat{e}}_{z}\right)-90^{\circ }|\rangle$$

(6) $$\langle \phi \rangle =\langle \mathrm{sgn}\left({\hat{d}}_{\mathrm{ND}}\cdot {\hat{e}}_{y}\right)\arccos\left[\frac{{\hat{d}}_{\mathrm{ND}}\cdot {\hat{e}}_{x}}{{\left({\hat{d}}_{\mathrm{ND}}\cdot {\hat{e}}_{x}\right)}^{2}+{\left({\hat{d}}_{\mathrm{ND}}\cdot {\hat{e}}_{y}\right)}^{2}}\right]\rangle$$

where ${\hat{d}}_{\mathrm{ND}}={\vec{d}}_{\mathrm{ND}}/\left|\right|{\vec{d}}_{\mathrm{ND}}\left|\right|$ is the unit vector defining the dimer orientation and $\left\{{\hat{e}}_{x},{\hat{e}}_{y},{\hat{e}}_{z}\right\}$ are canonical vectors oriented as the cartesian axes of the simulation box. With the values yielded by Equations (4)–(6) and their standard deviation, a clear picture of the average dimer orientation can be obtained. Notice that Equation (5) helps discern whether results from Equation (4) are caused by uniformly or randomly oriented particles. The calculation of the distribution profiles along *z* of both polar and azimuthal angles, detailed in Section S3, is especially useful to understand the relation between position and orientation.

Since our systems display a degree of orientational order, it makes sense to investigate whether the anisotropic particles (dimers) they host are also oriented along a preferential direction. To this end, we calculated the nematic order parameter $S_{2}$ and the nematic director $\hat{n}$ of particles, which, respectively, are the largest eigenvalue and the corresponding eigenvector of the following second-rank symmetric tensor [63]:

(7) $$Q=\frac{1}{2}\langle 3{\hat{d}}_{\mathrm{ND}}⊗{\hat{d}}_{\mathrm{ND}}-I\rangle$$

with ⊗ the dyadic product and I the second-rank unit tensor.

To characterise the dynamics of polymer chains and dimers, we calculated their translational and rotational diffusion coefficients. The former were obtained via the mean-squared displacement (MSD), defined as

(8) $${\langle \Delta r^{2}\left(t\right)\rangle }_{\alpha }={\langle |\vec{r}\left(t\right)-\vec{r}\left(0\right)|}^{2}\rangle_{\alpha }$$

Given the anisotropic nature of the lamellar phases, we also computed the MSD along *z* and in planes perpendicular to *z* (i.e., xy) [63] as reported, respectively, in Equations (9) and (10):

(9) $${\langle \Delta r_{z}^{2}\left(t\right)\rangle }_{\alpha }=\langle |\left[\vec{r}\left(t\right)-\vec{r}\left(0\right)\right]\cdot {\hat{e}}_{z}{{|}^{2}\rangle }_{\alpha }$$

(10) $${\langle \Delta r_{xy}^{2}\left(t\right)\rangle }_{\alpha }={\langle |\vec{r}\left(t\right)-\vec{r}\left(0\right)|}^{2}-|\left[\vec{r}\left(t\right)-\vec{r}\left(0\right)\right]\cdot {\hat{e}}_{z}{{|}^{2}\rangle }_{\alpha }$$

where $\vec{r}(t)$ is the position at time *t* and ${\langle ...\rangle }_{\alpha }$ stands for ensemble average over species α. Diffusion coefficients are proportional to the slope of the MSD at long times, where chains and NDs are supposed to have entered a diffusive regime. In particular:

(11) $$\begin{array}{c} D_{\alpha }=\lim_{t\to \infty }\frac{1}{6}\frac{\partial }{\partial t}{\langle \Delta r^{2}\left(t\right)\rangle }_{\alpha }\\ D_{z,\alpha }=\lim_{t\to \infty }\frac{1}{2}\frac{\partial }{\partial t}{\langle \Delta r_{z}^{2}\left(t\right)\rangle }_{\alpha }\\ D_{xy,\alpha }=\lim_{t\to \infty }\frac{1}{4}\frac{\partial }{\partial t}{\langle \Delta r_{xy}^{2}\left(t\right)\rangle }_{\alpha } \end{array}$$

To assess the dimers’ ability to orient and rotate with respect to the layers formed by the di-BCP, we calculated the rotational diffusion coefficient, defined as

(12) $$D_{\mathrm{rot},\mathrm{ND}}={\left[2\int_{0}^{\infty }dt\;\langle P_{1}\left[{\hat{d}}_{\mathrm{ND}}\left(t\right)\cdot {\hat{d}}_{\mathrm{ND}}\left(0\right)\right]\rangle \right]}^{-1}$$

where $P_{1}$ is the first-order Legendre polynomial [94,95]. This coefficient is relevant to ponder whether, at equilibrium, NDs are relatively free to rotate around their centre of mass or are perhaps constrained along a given direction, thus suggesting the existence of a preferential orientation.

## 3. Results and Discussion

**Figure 3:**
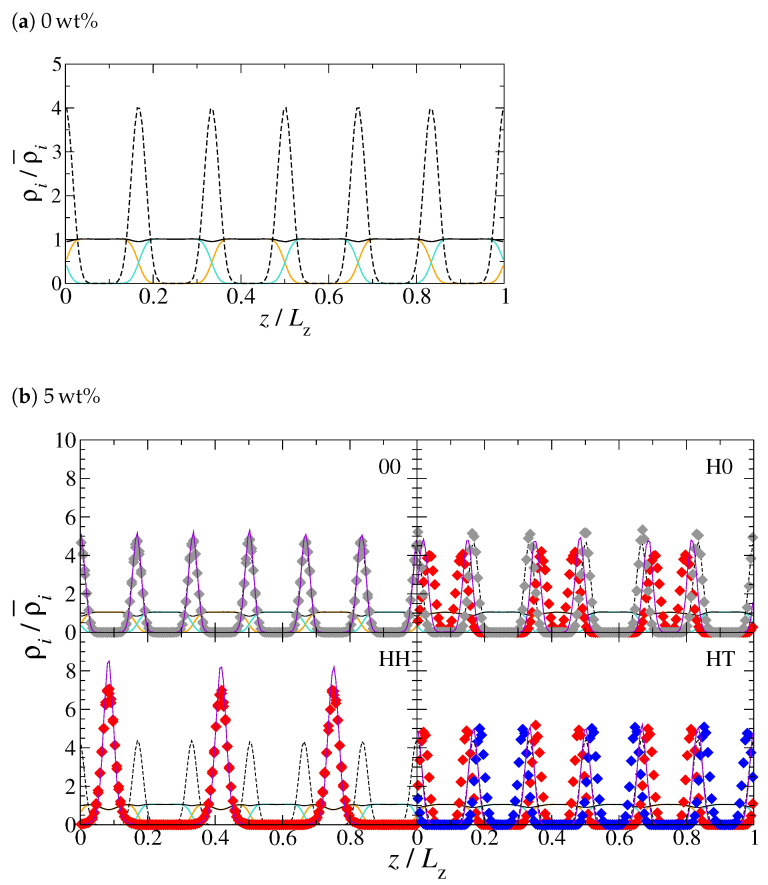
Density profiles of the pristine polymer (**a**) and the systems with 5 wt% particle mass fraction (**b**) in the z direction. The density profiles of h-block, t-block, chain beads and chains’ centre of mass are, respectively, indicated by orange, cyan, solid black and dashed black curves. The density profiles of H-like, T-like and neutral ND beads are represented as solid diamonds in red, blue and grey, respectively, and those of the NDs’ centre of mass as violet solid lines.

In this section, our aim is to discuss the dynamics of neutral and Janus NDs incorporated in lamellar phases of a di-BCP. To this end, aware of the relevance of the background structure in determining specific dynamical characteristics, we first report on the structural properties of our systems, with special focus on the NDs’ distribution and orientation within the di-BCP matrix. To start with, we examine the morphology of the pristine di-BCP. Its density profiles along the direction perpendicular to the lamellae are reported in Figure 3a and exhibit the typical periodicity of the copolymer blocks. As expected, the topological centres of the di-BCP chains are uniformly distributed within the inter-layer region (see also Section S2), where the total bead density (black solid line in Figure 3a is found to decrease slightly. Such a density reduction, while relatively soft, has in fact a crucial impact on the NDs’ distribution by providing some extra room to accommodate them.

**Figure 4:**
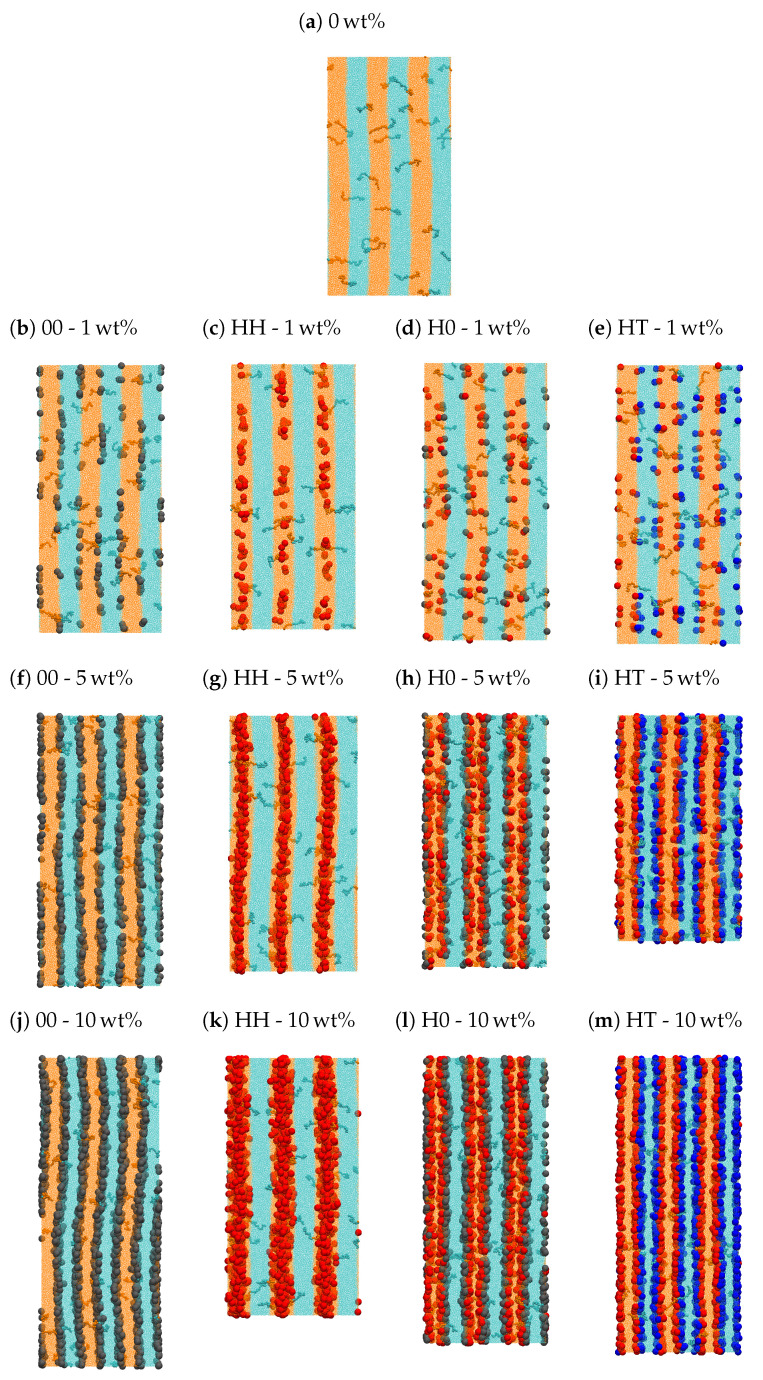
Frontal view of typical equilibrated configurations of the model PNCs studied in this work. NDs’ monomers are shown as spherical beads. Most polymer chains are depicted as points for clarity. However, some of them are magnified to better appreciate their conformations across the interface. Different colours indicate h-block (orange), t-block (cyan), neutral (grey), head-like (red) and tail-like (blue) monomers. Subfigures a to m are each identified by ND type and ND mass fraction, as detailed in Table 2.

The incorporation of NDs into this di-BCP does not destroy its smectic liquid-crystalline ordering, even at the largest nanoparticle loading considered here (10 wt%). A visual representation of the resulting PNCs as obtained after equilibration is reported in Figure 4 (a perspective view of the same configurations is also provided in Figure S4). As a general tendency, all types of NDs tend to arrange at the interfaces formed by the two polymer blocks, except HH-NDs, which are preferentially located within the h-block layers, as observed in Figure 4c,g,k at 1, 5 and 10 wt%, respectively. This can also be appreciated in the density profiles reported in Figure 3b for PNCs with 5 wt% of NDs and Figure S3 for all the PNCs studied here. While most NDs are constrained within the inter-layer region and thus display a quasi-two-dimensional distribution, HH-NDs tend to occupy the whole space available within the h-block, sufficiently far from the interface with the t-block, and their distribution assumes a three-dimensional character. This difference is especially evident at larger particle loading, as revealed by the broader base of peaks formed by HH-NDs in Figure S3c. We note that qualitatively similar tendencies were observed by Pine and co-workers, who investigated gold nanoparticles coated with polystyrene (PS) in poly(styrene-*b*-2 vinylpyridine) di-BCPs [96]. Although these nanoparticles exhibit a roughly spherical shape, they form preferential interactions with the PS block, similarly to our HH-NDs with the polymer *h* block. The authors found the nanoparticles mostly in the centre of the PS domains, with Gaussian distribution profiles whose width decreases as the PS domain size decreases or as the particle weight fraction increases. In principle, this behaviour would also be expected for 00-NDs, whose neutral interactions with both copolymer blocks should promote their homogeneous distribution throughout the mesophase, and for H0-NDs, which form favourable interactions with the h-block, where they should preferentially and uniformly disperse. In fact, this is not the case.

Figure 3b and Figure 4 and Figure S3 reveal that 00-NDs and H0-NDs are rather located at the h-block/t-block interfaces, suggesting the existence of entropic forces that, by offsetting those of an enthalpic origin, displace these NDs from the in-layer to the inter-layer domains. Enthalpy-driven forces are regulated by the intensity of the interactions between copolymer blocks and NDs, and are especially relevant when there is a significant affinity between these two species, as it is the case for HH-NDs and copolymer h-blocks. By contrast, entropy-driven forces are regulated by the volume available to the polymer chains and their subsequent ability to maximise the number of possible conformations. This can be achieved if the region between adjacent NDs, being excluded to the polymer chains, is minimised, resulting in an effective attraction between NDs, usually referred to as depletion attraction. Because thermodynamic equilibrium depends on the balance between these two contributions, if enthalpic forces are dominant, the ND/polymer contacts are maximised, whereas if entropic forces are dominant, these contacts are minimised. In almost all our model PNCs, the interactions between NDs and alike blocks are never dominant. Consequently, NDs are generally depleted from the polymer-rich domains and preferentially locate where the polymer density is lower, that is at the h-block/t-block interface. Functional NDs with an especially strong affinity with one copolymer block, that is HH-NDs, represent the only exception. However, it is sufficient to substitute one of the two H beads with a neutral bead (H0-NDs) to invert this tendency and re-establish the general behaviour. These preliminary considerations are necessary to understand what elements might enhance or hamper the mobility of both species, but they are not sufficient. Additional key factors are represented by NDs’ loading and space orientation.

Increasing the content of NDs from 1 to 10 wt% promotes the partial penetration of the NDs located at the interface into the adjacent layers. This effect is especially evident when separated inter-layer distributions of H0-NDs at 10 wt% enter in contact with each other, as observed in Figure 4l and density profiles of Figure 3b and Figure S3. Similarly, HH-NDs tend to homogeneously occupy the whole space available in the h-block regions and necessarily approach the interface. This can be appreciated by visual inspection of Figure 4 and by the analysis of Figure S3, where the NDs’ density distributions become broader and their peaks reduce upon increasing nanoparticle loading. Moreover, with the exception of PNCs comprising HH-NDs, the increase in particle load leads to a decrease in the di-BCP’s density at the interface. Although the lower chain density at the interface, observed in pristine polymers (see Figure 3a), does not stem from the presence of NDs, the latter indeed contribute to the re-arrangement of chain monomers at the interface. By contrast, HH-NDs create additional chain monomer density wells in the middle of h-block lamellae in addition to those already mentioned.

**Figure 5:**
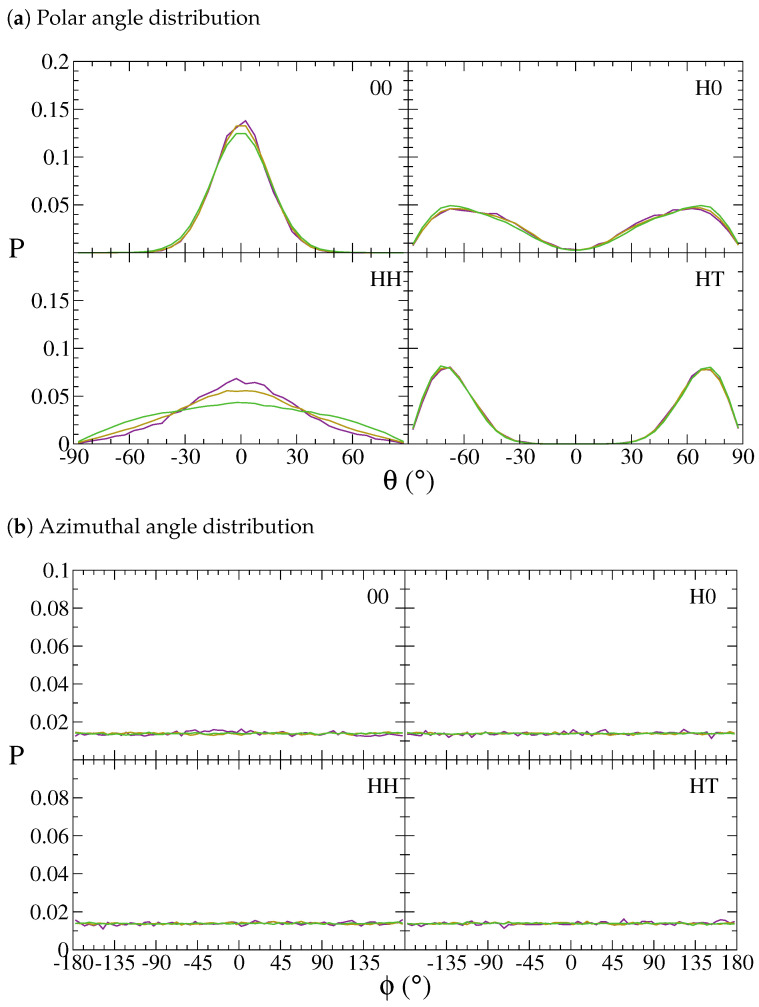
Probability distribution functions of polar (*θ*) and azimuthal (*ϕ*) ngles at particle mass fractions 1 wt% (purple lines), 5 wt% (yellow lines) and 10 wt% (green lines).

**Figure 6:**
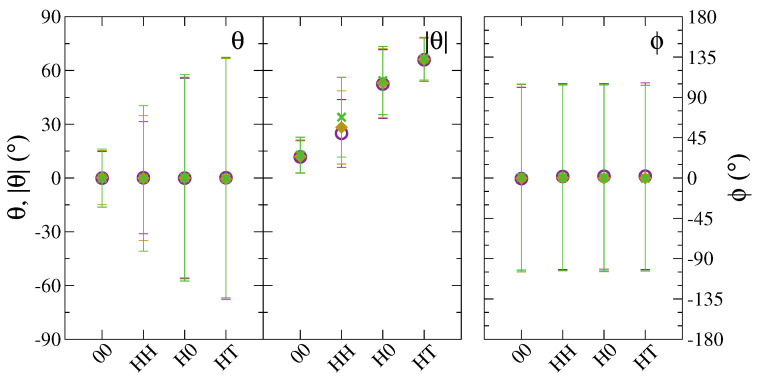
Polar (〈θ〉, 〈|θ|〉) and azimuthal (〈ϕ〉) average angle values of particles (00, H0, HH and HT) at mass fractions 1wt% (empty purple circles), 5wt% (yellow diamonds) and 10wt% (green crosses).

To assess the NDs’ orientation in the lamellar phases, we have determined the probability distributions of polar (θ) and azimuthal (ϕ) angles as well as their average values, respectively, shown in Figure 5 and Figure 6. In particular, θ is the angle between the ND longitudinal axis and its projection on the lamellar (xy) plane, whereas ϕ is the angle between this projection and a generic reference direction lying on the lamellar plane. The values of θ and |θ| indicate that both ND-chain interactions and interface topology have a relevant effect on the orientation of NDs, which, in contrast, is not especially influenced by the NDs’ loading. On the other hand, the azimuthal angle appears to be completely independent from both particle type and load. These observations suggest that there is not a preferential particle orientation within the xy plane but, depending on how NDs are functionalised, one can control their inclination with respect to this plane. Surprisingly, neutral NDs, which would be expected to exhibit random orientations and thus a uniform distribution of polar and azimuthal angles, are in fact almost completely constrained on the lamellar plane, as the relatively narrow Gaussian distributions of θ in Figure 5a and the small standard deviations of both θ and |θ| in Figure 6 suggest. This can also be appreciated in Figure S5, where the average value of these angles as a function of the ND position along the *z*-axis is reported. Less constrained appear to be HH-NDs, whose polar angles denote a more significant and broadly distributed inclination, thus suggesting a slightly larger propensity to explore more orientations than 00-NDs. Because of the periodic arrangement of h-block and t-block lamellae, the distribution of Janus NDs would also be expected to follow a similar pattern. This is indeed detected in the form of an alternating sign for θ in Figure S5. Such a sign alternation determines a split in the polar angle distributions of Figure 5a, which become bimodal, as opposed to the monomodal distributions generated by homogeneous NDs. These bimodal distributions are completely symmetric, as the left frame of Figure 6 indicates. The polar angle distributions, together with the values of |θ| in Figure 6 and the profiles in Figure S5 suggest that Janus NDs do not to lie on the lamellar plane, but display an inclination of at least $60^{\circ }$. Finally, the azimuthal angle, ϕ, does not show any relevant change with NDs’ interactions or content. Moreover, its average value and standard deviation are those typical of a uniform probability distribution, with no preferred orientation in the lamellar plane. The interested reader is referred to Section S4 and Table S1 for additional details on the properties of the continuous uniform probability distribution and the specific values of both polar and azimuthal angle distributions.

**Figure 7:**
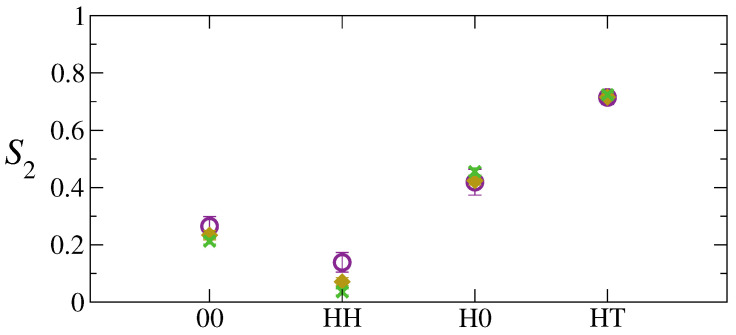
Nematic order parameter at mass fraction values 1wt% (empty purple circles), 5wt% (yellow diamonds) and 10wt% (green crosses).

To confirm these last observations on the orientation at the particle scale, we calculated the nematic order parameter, $S_{2}$, reported in Figure 7, and the average values of the nematic director components in the three space dimensions, shown in Table S2. We observe that homogeneous NDs, namely 00-NDs and HH-NDs, are very weakly ordered, with $S_{2}<0.3$. Janus NDs, and especially HT-NDs, display a more ordered arrangement, with significantly larger values of $S_{2}$, indicating a clear preference to be aligned along the *z*-direction, perpendicularly to the lamellar planes. The nematic order parameter is practically independent from the particle loading, being the differences observed among 1, 5 and 10 wt% in Figure 7 safely negligible.

**Figure 8:**
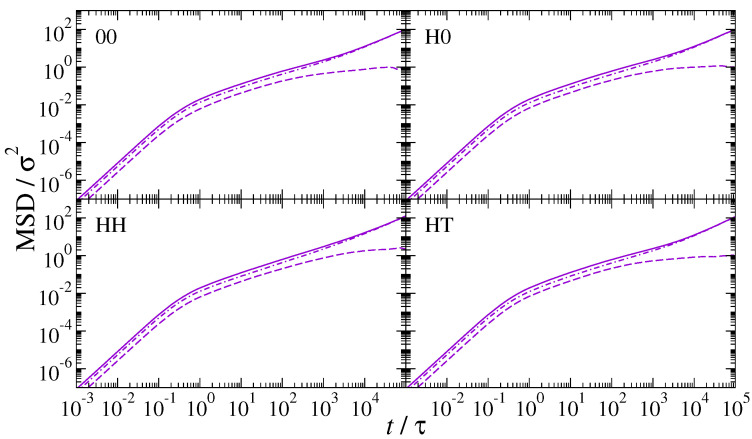
Mean squared displacement of neutral and Janus NDs at 5 wt% in 3D-space (continuous lines), within the lamellar xy plane (dashed-dotted lines) and along the *z* direction (dashed lines).

In the light of these considerations on positional and orientational distribution of our model NDs, we now discuss the dynamical properties of the systems studied. To this end, we have first calculated the MSD of polymer chains and NDs. The latter are reported in Figure 8 and the interested reader is referred to Figure S6 for the polymer chains’ MSD. As a general tendency, the MSD of the di-BCP chains are not especially affected by the specific nature of NDs or their content. In particular, the chains dynamics in the lamellar xy plane exhibits a diffusive behaviour at long-time scales, which follows a moderate cage-effect imposed by neighbouring particles and chains. In the *z* direction, perpendicular to the lamellae, the chain dynamics is much slower and never achieves the long-time diffusive regime within our simulations (eight time decades). NDs display analogous anisotropic dynamics with very similar trends in their MSDs across the time scales. Only PNCs with a small number of HH-NDs, the only NDs that are located within the lamellar layers, show slightly faster dynamics, with the MSD at long times approximately twice as large as that of other NDs (see Figure S7a). We believe that such a larger mobility is due to the space available to HH-NDs to diffuse, which is larger than the quasi-2D interface region where the other NDs are constrained to move. This difference becomes less and less relevant at increasing ND loading.

**Figure 9:**
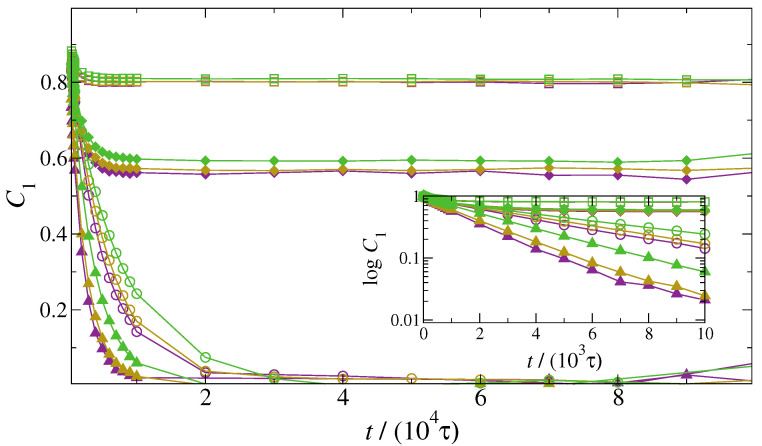
Time autocorrelation functions of the orientation of NDs. The inset provides a zoom at short time scales (notice the log-lin scale). Systems containing 00 (empty circles), HH (solid triangles), H0 (solid diamonds) and HT (empty squares) NDs are shown at particle mass fractions 1wt% (purple), 5wt% (yellow) and 10wt% (green).

In addition to the translational dynamics, we also investigated the NDs’ ability of reorienting over time, which is especially important to understand to what extent NDs can be responsive to thermal fluctuations and external stimuli. To this end, we calculated the time autocorrelation functions of ND orientation, shown in Figure 9. Janus NDs display very strong correlations, approaching an asymptotic value at relatively short times and suggesting a rather constrained rotational dynamics that does not achieve the diffusive regime. By contrast, 00-NDs and HH-NDs display a very different behaviour, with a fast decay of $C_{1}$ to zero at relatively short time scales, denoting the full achievement of the diffusive regime. While the functionalisation of NDs induces relevant changes in the rotational dynamics, the impact of ND loading is practically negligible, further confirming that specific dynamical properties can be achieved at very low nanoparticle loading.

**Figure 10:**
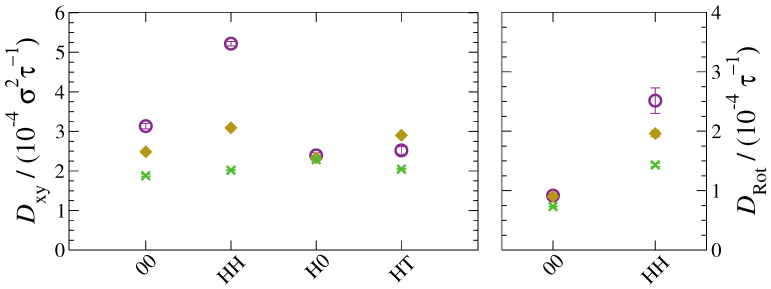
Translational diffusivity of NDs in the plane (**left**) and rotational diffusivity (**right**) for various particle mass fractions: 1wt% (purple empty circles), 5wt% (yellow full diamonds) and 10wt% (green crosses).

From the NDs’ MSD and orientational autocorrelation functions, the translational and rotational diffusion coefficients were, respectively, determined. The former were obtained by applying Equation (10) at long times, that is when the diffusive regime was reached and MSD ∝t. This calculation is only possible in the lamellar (xy) plane as no diffusion regime is achieved in the *z* direction. Rotational diffusivities were calculated as the inverse of the integrated autocorrelation functions, according to Equation (12). The integral curves of the autocorrelation functions together with the convergence behaviour of $D_{\mathrm{Rot},\mathrm{ND}}$ are displayed in Figure S8. These curves evidence the convergence of diffusivities of homogeneous NDs and their divergence in the case of Janus NDs. In other words, no rotational diffusion coefficients could be obtained for H0-NDs and HT-NDs because no diffusive regime is achieved. The so-calculated translational and rotational diffusivities are shown, respectively, in the left and right frames of Figure 10. Interestingly, translational diffusivities, $D_{xy}$, which assume values approximately between 2 and $5\times 10^{-4}\sigma^{2}\tau^{-1}$, do not seem to depend significantly on particle load, except in PNCs incorporating HH-NDs. We know that these dimers are preferentially concentrated in the core of the h-block domains, which are significantly larger than the quasi-two-dimensional inter-layer spacings hosting neutral or Janus NDs. Consequently, the number of HH-NDs per unit volume is comparatively low and $D_{xy}$ significantly larger. However, upon increasing ND loading, this difference gradually diminishes and becomes practically negligible at 10 wt%, where $D_{xy}\approx 2\times 10^{-4}\sigma^{2}\tau^{-1}$ for all particle species. Rotational diffusivities are shown for those NDs whose integrated autocorrelation functions converged and a diffusive regime is actually achieved, i.e., homogeneous NDs only. It seems that rotational diffusivities are affected by both interactions and particle content. Specifically, HH-NDs show faster rotational dynamics than 00-NDs for all ND mass fractions, with lower ND load producing larger rotational diffusivities. In the case of 00-NDs, changing particle mass fraction does not have any relevant effect on the rotational diffusivity. We believe that this is most likely due to similar considerations to those valid for translational diffusion: while 00-NDs are constrained in the inter-layer region and show a very narrow distribution of planar angles, HH-NDs can rotate in a significantly larger volume allowing for a much broader distribution of angular inclinations.

## 4. Conclusions

In summary, we investigated the structural and dynamical properties of PNCs of di-BCP chains assembled in lamellar structures. These amphiphilic polymers are particularly attractive because they can self-assemble into liquid-crystalline mesophases, providing a long-ranged ordered pattern for the controlled position and orientation of guest particles. It is generally accepted that controlling the distribution of NPs in a polymer matrix can be achieved by chemically modifying their surface and thus enhancing their affinity with the copolymer. Nevertheless, we showed here that pure entropy-driven forces are indeed able to position NPs in specific domains regardless of whether they form strong enthalpic interactions with either copolymer block. In particular, four distinct types of NDs (two homogeneous and two Janus-like) at different mass fractions were studied. Anisotropic particles with a uniaxial symmetry, such as NDs, have two additional degrees of freedom that characterise their orientation in space. Understanding how to orient functional NPs in a polymer is crucial for a number of industry-relevant applications. We showed that, by accurately tuning the balance between enthalpic and entropic contributions, it is indeed possible to control orientation as well as position of NDs in a lamellar phase of a di-BCP. In general, NDs tend to concentrate in the inter-layer spacing separating the two copolymer blocks. However, this was not the case for NDs forming especially strong interactions with one of the two copolymer blocks, which are accommodated far from the inter-layer spacing. Moreover, while homogeneous NDs are preferentially lying on planes parallel to the lamellae, either in the core of the copolymer block or at the interface between two distinct copolymer blocks, Janus NDs are significantly tilted, with their longitudinal axis almost perpendicular to the lamellae. These structural properties determine the dynamics of both NDs and polymer chains. Here, we specifically focused on the dynamics of the former species and estimated their mobility by calculating translational and rotational diffusion coefficients in planes xy parallel to the lamellae and in the *z* direction perpendicular to them. While all NDs achieve the long-time diffusive regime in xy planes, none of them are found to diffuse in the *z* direction, basically being constrained in smectic layers or inter-layers. Similarly, Janus NDs display a very limited rotational dynamics as compared to that of homogeneous NDs, unveiling strong angular correlations that persist over time. Considering the little attention that PNCs incorporating Janus NDs have thus far received, we hope that our contribution will be able to stimulate further experimental work and solid ground to corroborate our predictions.

## Figures and Tables

**Table 1:** Force-field parameters employed in Equations (1) and (2), respectively, describing the non-bonded and bonded interactions of h-block and t-block chain beads and H, T and 0-ND beads. For all interactions, $r_{c}=2^{1/6}\;\sigma$. All parameters are invariant under subindex inversion ($A_{\alpha \beta }=A_{\beta \alpha }$).

Bead		Non-Bonded		Bonded		
α	β	$\epsilon_{\alpha \beta }/\epsilon$	$\Delta_{\alpha \beta }/\sigma$	$K_{\alpha \beta }/\epsilon \sigma^{-2}$	$R_{0,\alpha \beta }/\sigma$	$\sigma_{\alpha \beta }/\sigma$
h	h	1	0	30	1.5	1
h	t	52	0	30	1.5	1
t	t	1	0	30	1.5	1
H	H	52	2	15	3.0	3
H	T	52	2	15	3.0	3
H	0	52	2	15	3.0	3
T	T	52	2	15	3.0	3
T	0	52	2	15	3.0	3
0	0	52	2	15	3.0	3
H	h	1	1			
H	t	52	1			
T	h	52	1			
T	t	1	1			
0	h	1	1			
0	t	1	1			

**Table 2:** Summary of systems studied. Information includes the system id number, the number of di-BCP chains (*n*), ND type, the mass fraction of NDs (w/wt%) and the total number of NDs ($n_{\mathrm{ND}}$).

System	*n*	ND Type	w/wt%	$n_{\mathrm{ND}}$
PNC${}_{0}$	12,696		0	0
PNC${}_{1}$	12,696	00	1	96
PNC${}_{2}$	12,696	HH	1	96
PNC${}_{3}$	12,696	H0	1	96
PNC${}_{4}$	12,696	HT	1	96
PNC${}_{5}$	12,696	00	5	510
PNC${}_{6}$	12,696	HH	5	510
PNC${}_{7}$	12,696	H0	5	510
PNC${}_{8}$	12,696	HT	5	510
PNC${}_{9}$	12,696	00	10	1080
PNC${}_{10}$	12,696	HH	10	1080
PNC${}_{11}$	12,696	H0	10	1080
PNC${}_{12}$	12,696	HT	10	1080

## Data Availability

The data presented in this study are openly available in Zenodo at 10.5281/zenodo.4665530 and 10.5281/zenodo.4666523 [97,98]. The software used to produce final data from trajectories is available at 10.5281/zenodo.4660283 [93].

## Supplementary Materials

The following material is available online at https://www.mdpi.com/article/10.3390/polym13091524/s1, Section S1: Dimer mass calculation (including Equation (S1)), Section S2: Setup and Equilibration (including Figure S1: Two possible initial lamellar arrangements, Figure S2: Initial configurations), Section S3: Calculation of ND orientation vs. position in the simulation box (including Equations (S2) to (S4)), Section S4: The continuous uniform distribution (including Equation (S5), Table S1: Rectangular distribution data for polar and azimuthal angles), and, additionally, Table S2: Nematic order parameter and nematic director components for NDs in each system, Figure S3: Density profiles, Figure S4: Polar, absolute polar, and azimuthal average angle values as a function of the *z* coordinate, Figure S5: Mean squared displacement of chain monomers and chains’ centre of mass at each system, Figure S6: Mean squared displacement of NDs at each system, Figure S7: Accumulated time autocorrelation functions and convergence behaviour of $D_{\mathrm{Rot}}$ as a function of time.

## Abbreviations

The following abbreviations are used in this manuscript:

PNC	Polymer nanocomposite
NP	Nanoparticle
BCP	Block copolymer
ND	Nano-dimer
MD	Molecular Dynamics
CG	Coarse-grain(ed)
LJ	Lennard–Jones (potential)
WCA	Weeks–Chandler–Andersen (potential)
FENE	Finitely Extensible Non-linear Elastic (potential)
NPT	Isothermal-isobaric or constant *N*, *P* and *T* (ensemble)
MSD	Mean-squared displacement
