# Supplementary material for: Molecular Dynamics of Janus Nanodimers Dispersed in Lamellar Phases of a Block Copolymer
Source: Polymers (Basel). 2021 May 9;13(9):1524. doi: 10.3390/polym13091524 (PMC8126030; doi:10.3390/polym13091524)
Supplement: Supplementary file 1 [file polymers-13-01524-s001.zip › polymers-1197191-supplementary.pdf]

Supplementary Materials: Molecular Dynamics of Janus Nanodimers Dispersed in Lamellar Phases of a Block Copolymer

J. Javier Burgos-Mármol [1] and Alessandro Patti [2,*]

S1. Dimer mass calculation

To estimate the mass of the dimers, we took into account the following conditions: (*i*) the mass of beads scales with their volume (i.e. with the cube of the diameter); (*ii*) relatively large nanoparticles can result from the aggregation of smaller clusters whose packing determines their final density; and (*iii*) there is a degree of overlap between the dimer beads. The first condition implies that if $\sigma_\beta = 3\sigma_\alpha$, then $m_\beta = 27m_\alpha$. The second condition amends the first one in case of large particles, whose mass should be modified by the packing fraction. We have arbitrarily chosen the largest possible packing fraction, that of a Face Centred Cubic (FCC) unit cell, being $\phi_{\max} = \pi/(3\sqrt{2}) \approx 0.74$ [89,90]. Finally, the third condition is a consequence of the model described in Section 2.1. More specifically, while the diameter of the ND beads is $\sigma_\mathrm{H} = \sigma_\mathrm{T} = \sigma_0 = 3\,\sigma$, the distance at the minimum of the ND stretching potential defined in Equation (2) is $d_\mathrm{ND} \approx 2.5\,\sigma$, i.e. the equilibrium bond length between the two components of the dimer is about 17% shorter than the hard-sphere distance between them. This lens-shaped overlapping volume is equivalent to the sum of the volume of two spherical caps [91], and it reads:[92]

$$V_\mathrm{lens} = \frac{\pi}{12 d_{\alpha\beta}} \left(\frac{\sigma_\alpha}{2} + \frac{\sigma_\beta}{2} - d_{\alpha\beta} \right)^2 \left[d_{\alpha\beta}^2 + d_{\alpha\beta}(\sigma_\alpha + \sigma_\beta) - \frac{3}{4}(\sigma_\alpha - \sigma_\beta)^2 \right], \tag{S1}$$

which, for $\sigma_\alpha = \sigma_\beta = 3\,\sigma$ and $d_{\alpha\beta} = 2.5\,\sigma$, is reduced to $V_\mathrm{lens,ND} \approx 0.56\,\sigma^3$, equivalent to $\approx 2\,\%$ of the total volume of the dimer. Consequently, after all the conditions are imposed, the final mass of a ND bead results to be $m_\mathrm{H} = m_\mathrm{T} = m_0 = 19.59\,\mathrm{M}$.

We note that the third condition could also be applied to account for the overlapping of consecutive beads in a polymer chain. By substituting $\sigma_\alpha = \sigma_\beta = \sigma$ and $d_{\alpha\beta} \approx 0.96\,\sigma$ (the polymer's equilibrium bond length from Equation (2), the overlapping volume is $V_\mathrm{lens,chain} \approx 0.0012\,\sigma^3$, which is approximately 0.1 % of the volume of the two beads and thus safely negligible.

S2. Setup and Equilibration

Initial configurations were created by first allocating chains. Initially, three chains were grown along the z direction (arbitrarily selected) at the same x, y coordinates. Instead of replicating this set of three chains, a shift of $\Delta x = 2^{1/6}\,\sigma$ and $\Delta z = l_\mathrm{h}\,\sigma$ is applied to create a second row by growing three additional chains with opposite interactions next to the first row, making the centre of the chains of the second row by half-chain length with respect to those in the first row. The aim of this shift in the centres of mass is to distribute them equitatively in all the inter-layer planes. Subsequently, the two initial rows were replicated in the x direction and then the resulting sheet was replicated in the y direction until the final number of chains was achieved. Due to the repulsive potential used in this study (see Equation (1) and Table 1), producing alternating chain-ends-only and centres-only interphases would have created sets of dynamically almost-independent lamellae. Figure S1 shows the difference between both cases, where systems are shown as diblock copolymer sheets piled on top of each other.

Nanodimers were positioned with the intention of minimising the number of defects and generating systems as homogeneously distributed as possible, with initial positions

(a) n-htth **(b)** n-htht

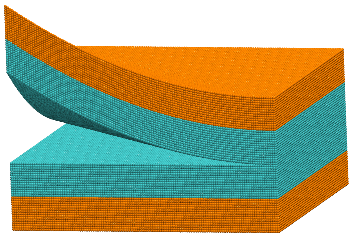

Figure S1. Two possible initial lamellar arrangements. While (a) produces a homogeneous distribution of chain centres and ends in every inter-layer plane, (b) produces alternating ending beads-only and central beads-only interfaces.

reported in Figure S2. Particles can be seen allocated between regularly spaced polymer sheets and with their centre of mass aligned with the inter-layer planes.

(a) 0 wt% **(b)** 00 - 1 wt% **(c)** 00 - 5 wt% **(d)** 00 - 10 wt%

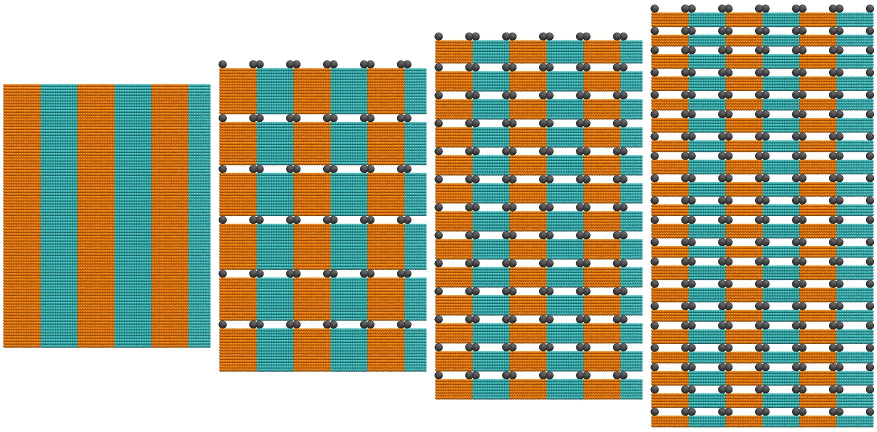

Figure S2. Initial configurations employed to equilibrate the systems studied in this work. Interactions were changed after thermalisation and pressurisation of these configurations.

For the pure copolymer, we calculated the equation of state at $T = 1\epsilon/k_B$ expanding the system between $P_1 = 1.5\,\epsilon\sigma^{-3}$ and $P_2 = 150\,\epsilon\sigma^{-3}$ and subsequently compressing it back to the initial pressure. Two expansion-compression cycles, differing by the pressure rate applied, were performed. In particular, the slow and fast cycles were respectively run at $|\delta P/\delta t| = 3.0 \cdot 10^{-3}\,\epsilon\sigma^{-3}\tau^{-1}$ and $|\delta P/\delta t| = 1.5 \cdot 10^{-2}\,\epsilon\sigma^{-3}\tau^{-1}$. From the resulting equation of state, reported in Figure 2, we selected $P = 11.3\,\epsilon\sigma^{-3}$ to equilibrate a stable lamellar phase at density $\rho = 1.0\,\sigma^{-3}$.

For nanocomposites, thermalisation and pressurisation run for $t = 5 \cdot 10^4\,\delta t$ from $T = 0$ to $T = 1.0\,\epsilon/k_B$ at $P = 11.3\,\epsilon\sigma^{-3}$ in the NPT ensemble. Following this short run, chains filled the empty spaces, but had no time to rearrange to destroy the pre-ordered structure when the density was still relatively low and lamellae could melt. This process was performed with 00-NDs only, but was used as the starting point for equilibration of all systems containing particles. The non 00-NDs would have their interactions activated before equilibration. Once all systems were at target pressure and temperature, equilibration run for $t = 2 \cdot 10^8\,\delta t$ in the NPT ensemble, to stabilise total energy E, volume V_{box}, and box lengths.

S3. Calculation of ND orientation *vs* position in the simulation box

We employed the same bin-segmentation used to calculate the density profiles and determined the orientation by measuring the polar angle $\theta \in [-90^\circ, 90^\circ]$, its absolute value, and the azimuthal angle $\phi \in [-180^\circ, 180^\circ)$. More specifically:

$$\langle \theta(z_i \pm \delta z) \rangle_i = \langle \arccos\left(\hat{\boldsymbol{d}}_{\mathrm{ND}} \cdot \hat{\boldsymbol{e}}_{\mathrm{z}}\right) - 90^\circ \rangle_i \tag{S2}$$

$$\langle |\theta(z_i \pm \delta z)| \rangle_i = \langle |\arccos\left(\hat{\boldsymbol{d}}_{\mathrm{ND}} \cdot \hat{\boldsymbol{e}}_{\mathrm{z}}\right) - 90^\circ| \rangle_i \tag{S3}$$

$$\langle \phi(z_i \pm \delta z) \rangle_i = \left\langle \mathrm{sgn}(\hat{\boldsymbol{d}}_{\mathrm{ND}} \cdot \hat{\boldsymbol{e}}_{\mathrm{y}}) \arccos\left[\frac{\hat{\boldsymbol{d}}_{\mathrm{ND}} \cdot \hat{\boldsymbol{e}}_{\mathrm{x}}}{(\hat{\boldsymbol{d}}_{\mathrm{ND}} \cdot \hat{\boldsymbol{e}}_{\mathrm{x}})^2 + (\hat{\boldsymbol{d}}_{\mathrm{ND}} \cdot \hat{\boldsymbol{e}}_{\mathrm{y}})^2} \right] \right\rangle_i \tag{S4}$$

where $\hat{\boldsymbol{d}}_{\mathrm{ND}} = \vec{\boldsymbol{d}}_{\mathrm{ND}} / ||\vec{\boldsymbol{d}}_{\mathrm{ND}}||$ is the unit vector defining the dimer orientation, $\{\hat{\boldsymbol{e}}_{\mathrm{x}}, \hat{\boldsymbol{e}}_{\mathrm{y}}, \hat{\boldsymbol{e}}_{\mathrm{z}}\}$ are canonical vectors oriented as the cartesian axes of the simulation box, and $\langle ... \rangle_i$ is the ensemble average in bin i. These plots are shown in Figure S5.

S4. The continuous uniform distribution

This distribution is a rectangular Probability distribution function (PDF), meaning that all values within the interval are equiprobable:

$$\mathcal{U}(a, b) = \frac{1}{b - a} \tag{S5}$$

where a and b are the limits of the distribution. This distribution has a mean value $\mu_{\mathcal{U}} = (b + a)/2$ and a standard deviation $\sigma_{\mathcal{U}} = |b - a|/\sqrt{12}$. Below, the properties of this distribution when applied to polar and azimuthal angles.

Table S1. Rectangular distribution data for polar and azimuthal angles.

Angle	PDF	$\mu_{\mathcal{U}}$	$\sigma_{\mathcal{U}}$
θ	$\mathcal{U}_\theta(-90^\circ, 90^\circ)$	0°	52°
$\|\theta\|$	$\mathcal{U}_{\|\theta\|}(0^\circ, 90^\circ)$	45°	26°
ϕ	$\mathcal{U}_\phi(-180^\circ, 180^\circ)$	0°	104°

S5. Additional data

Table S2. Nematic order parameter and nematic director components for NDs in each system.

System	S_2	$\langle n_x \rangle$	$\langle n_y \rangle$	$\langle n_z \rangle$
PNC_1	0.265 ± 0.033	0.48 ± 0.55	0.39 ± 0.56	-0.006 ± 0.035
PNC_2	0.139 ± 0.034	0.45 ± 0.55	0.45 ± 0.53	0.00 ± 0.13
PNC_3	0.419 ± 0.045	-0.003 ± 0.081	-0.005 ± 0.067	0.9944 ± 0.0063
PNC_4	0.715 ± 0.025	-0.004 ± 0.035	-0.002 ± 0.038	0.9986 ± 0.0015
PNC_5	0.234 ± 0.016	0.52 ± 0.51	0.44 ± 0.53	0.000 ± 0.016
PNC_6	0.071 ± 0.014	0.51 ± 0.50	0.48 ± 0.49	0.00 ± 0.12
PNC_7	0.424 ± 0.016	-0.003 ± 0.028	-0.005 ± 0.030	0.99913 ± 0.00091
PNC_8	0.7156 ± 0.0096	0.000 ± 0.016	0.000 ± 0.016	0.99974 ± 0.00028
PNC_9	0.213 ± 0.010	0.57 ± 0.53	0.24 ± 0.59	0.000 ± 0.013
PNC_{10}	0.036 ± 0.011	-0.02 ± 0.32	0.08 ± 0.28	0.84 ± 0.34
PNC_{11}	0.455 ± 0.012	0.000 ± 0.020	-0.004 ± 0.019	0.99961 ± 0.00036
PNC_{12}	0.7239 ± 0.0077	-0.001 ± 0.011	-0.001 ± 0.010	0.99989 ± 0.00011

S6. Additional figures

(a) 1 wt%

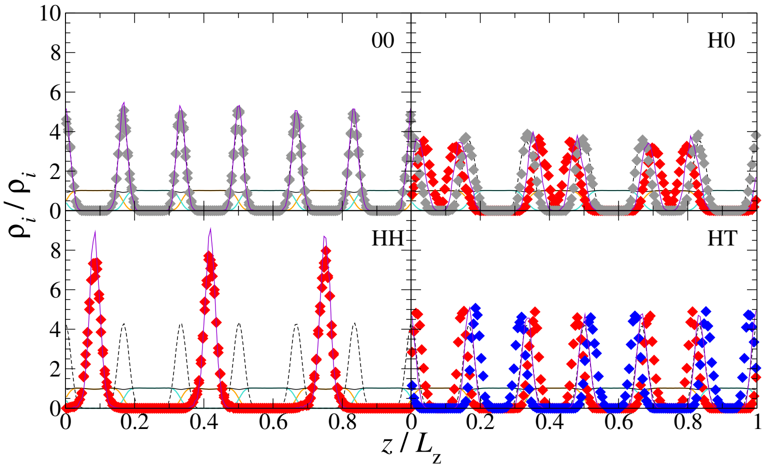

(b) 5 wt%

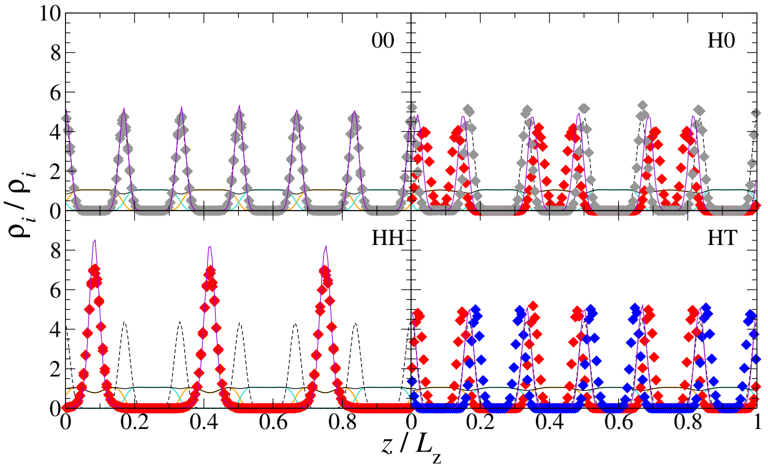

(c) 10 wt%

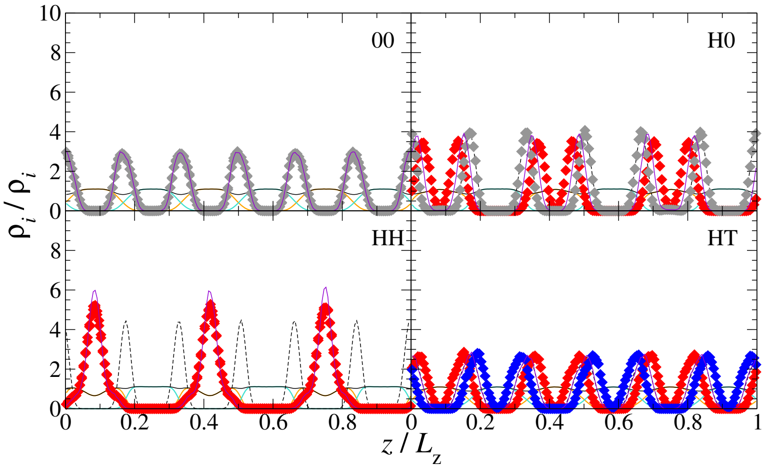

Figure S3. Density profiles in the z direction of h-block (orange line), t-block (cyan line) and both chain beads (black line); chains' centre of mass (black dashed line); particles' H-like (red diamonds), T-like (blue diamonds), neutral (grey diamonds) ND beads; and particles' centre of mass (violet line).

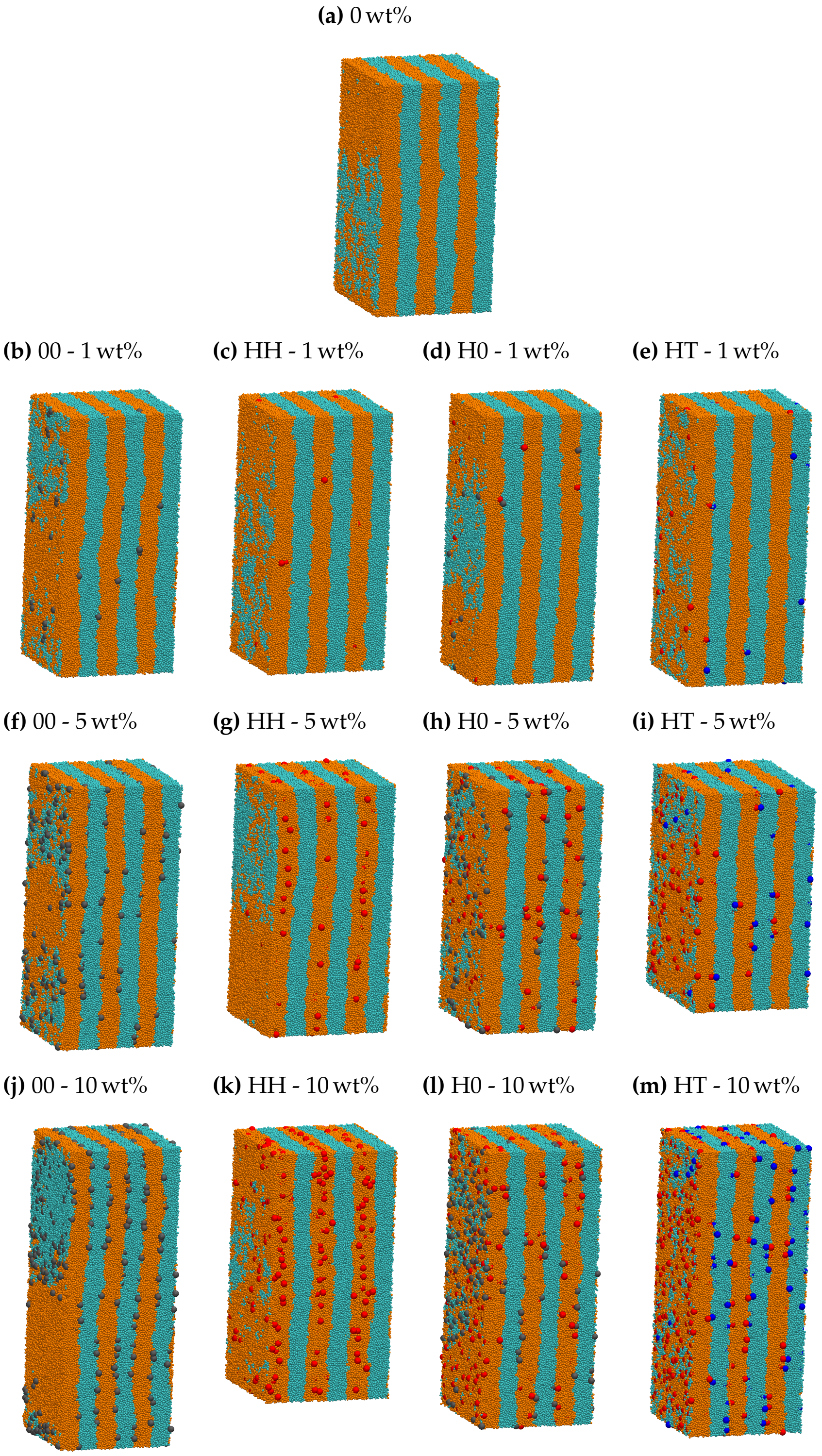

Figure S4. Perspective view of typical equilibrated configurations of the model PNCs studied in this work. NDs and chains' monomers are shown as spherical beads. Different colours indicate h-block (orange), t-block (cyan), neutral (grey), head-like (red) and tail-like (blue) monomers. Subfigures a to m are each identified by ND type and ND mass fraction, as detailed in Table 2.

(a) 1 wt%

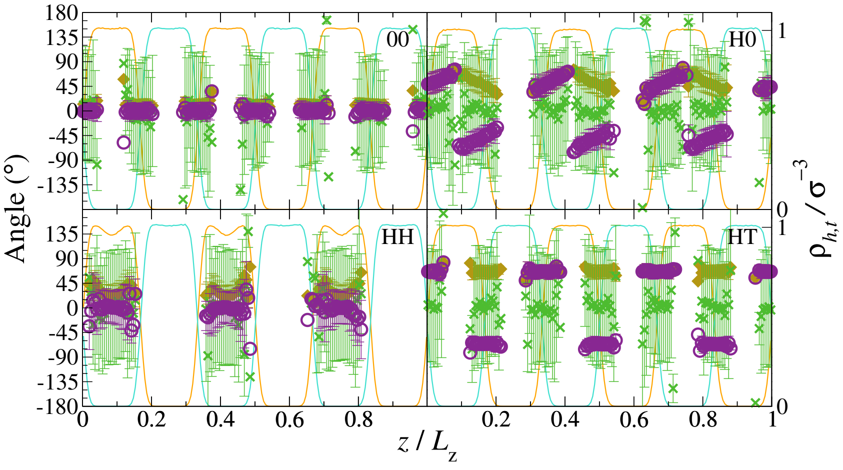

(b) 5 wt%

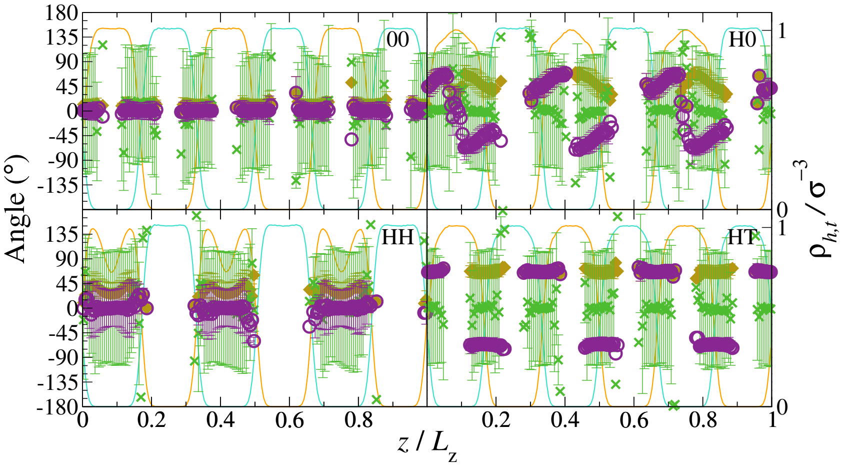

(c) 10 wt%

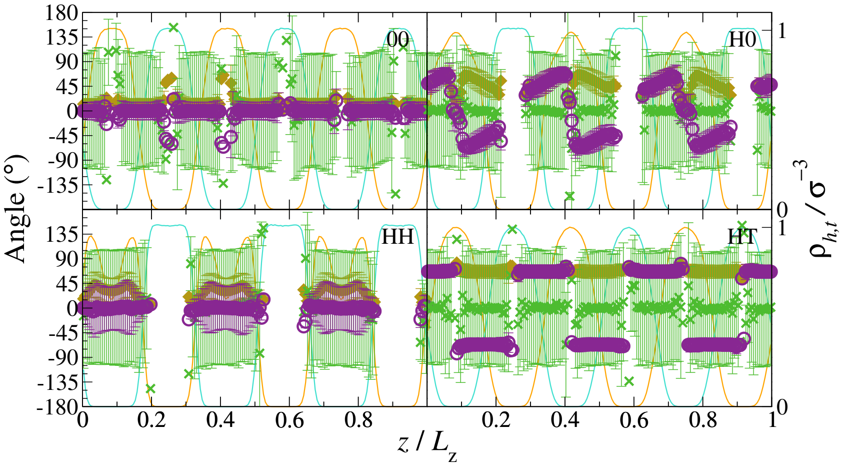

Figure S5. Polar $\langle\theta\rangle$ (purple empty circles), absolute polar $\langle|\theta|\rangle$ (yellow full diamonds) and azimuthal $\langle\phi\rangle$ (green crosses) average angle values as a function of the z coordinate. As a reference, orange and cyan lines are the local density of h-block and t-block chain beads, respectively.

(a) 1 wt%

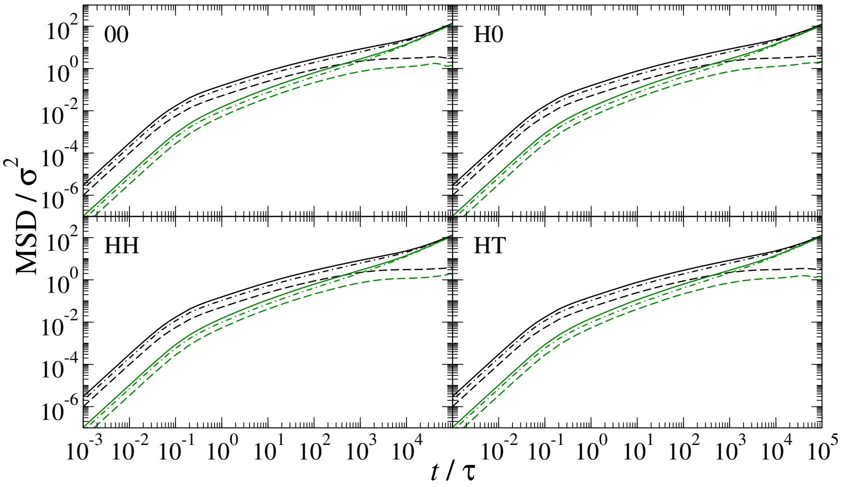

(b) 5 wt%

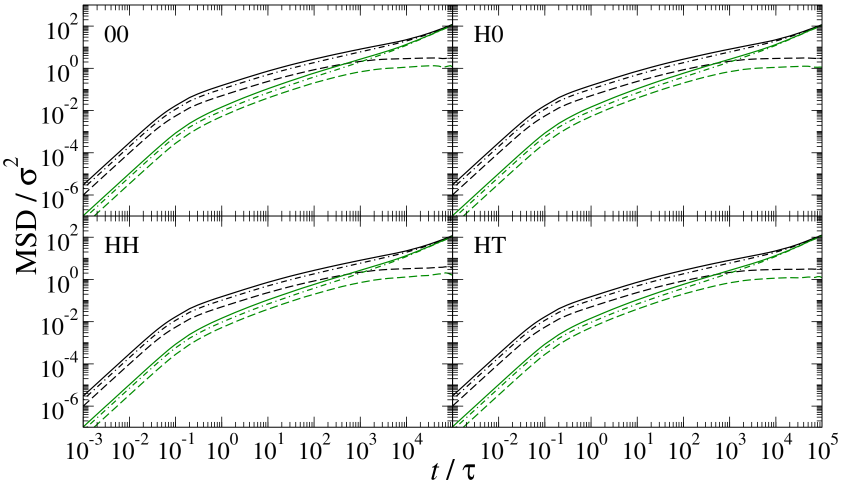

(c) 10 wt%

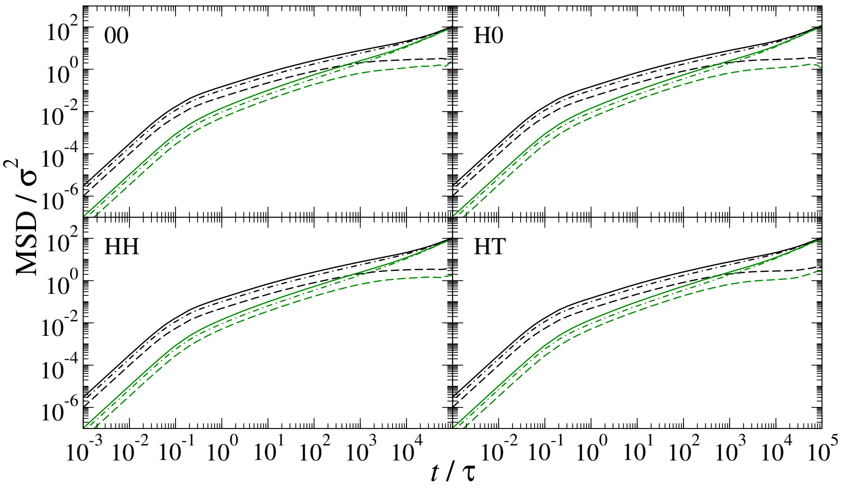

Figure S6. Mean squared displacement of chain monomers (black lines) and chains' centre of mass (green lines) at each system. MSD is shown for three-dimensional trajectories (continuous lines), movement in the lamellar plane (dotted-dashed lines) and movement along z-axis (dashed lines).

(a) 1 wt%

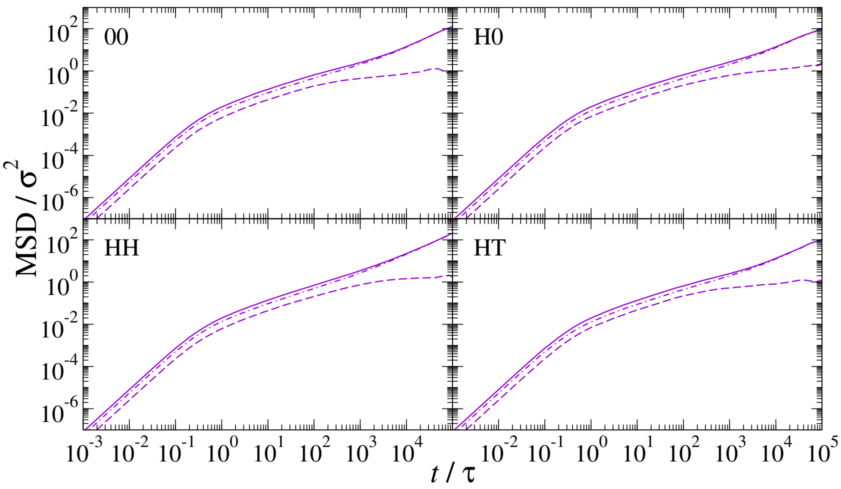

(b) 5 wt%

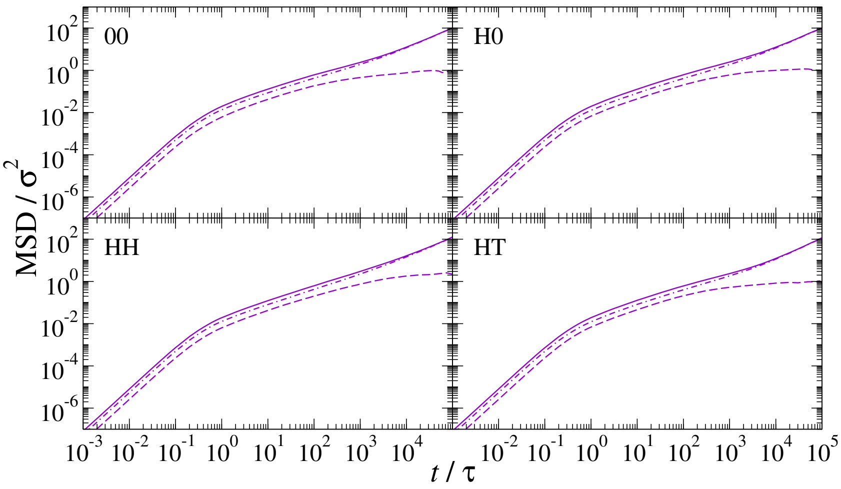

(c) 10 wt%

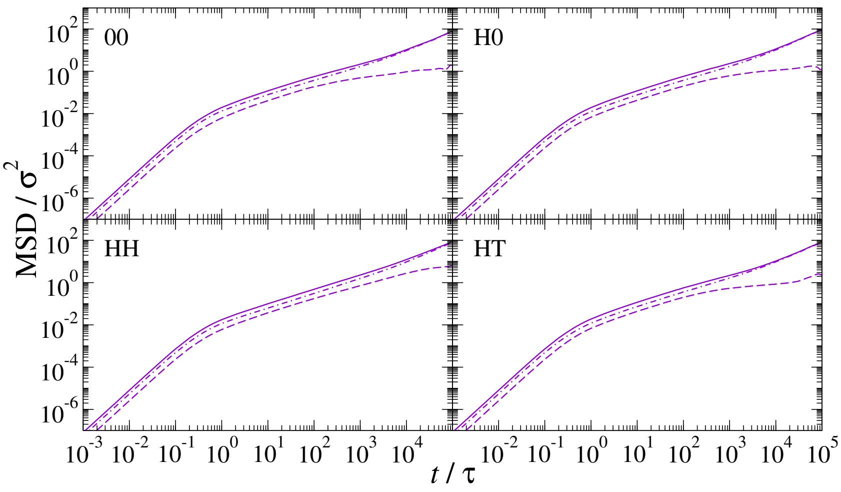

Figure S7. Mean squared displacement of NDs (violet lines) at each system. MSD is shown for three-dimensional trajectories (continuous lines), movement in the lamellar plane (dotted-dashed lines) and movement along z-axis (dashed lines).

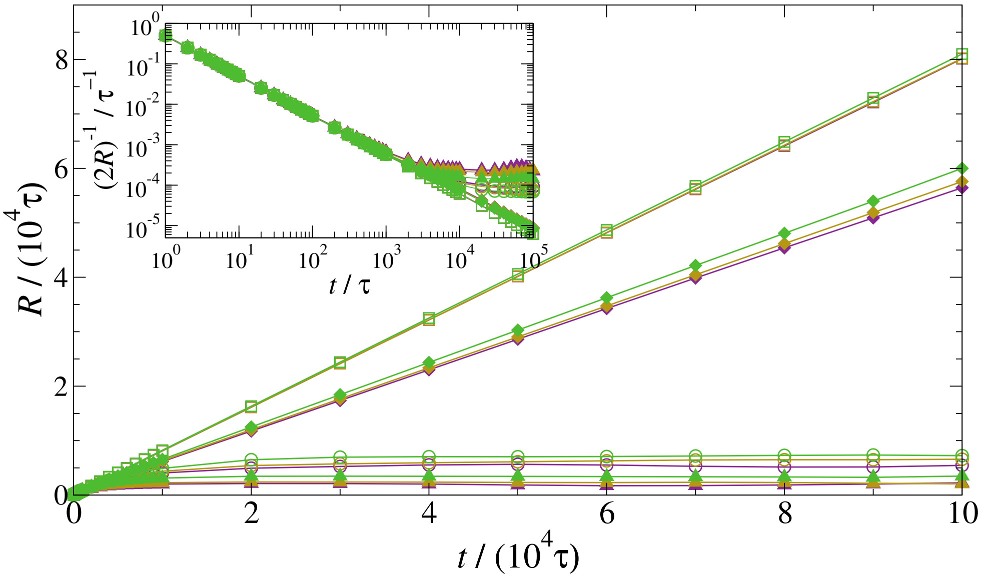

Figure S8. Accumulated time autocorrelation functions $R \equiv \int_0^t \mathrm{d}t' \, \langle P_1[\hat{\boldsymbol{d}}_{\mathrm{ND}}(t') \cdot \hat{\boldsymbol{d}}_{\mathrm{ND}}(0)] \rangle$ (main frame), and convergence behaviour of D_{Rot} as a function of time (inset). Systems containing 00 (empty circles), HH (full triangles), H0 (full diamonds) and HT (empty squares) NDs are shown at particle mass fractions 1 wt% (purple), 5 wt% (yellow) and 10 wt% (green).

References

1. Yang, Q.; Loos, K. Janus nanoparticles inside polymeric materials: interfacial arrangement toward functional hybrid materials. *Polym. Chem.* **2017**, *8*, 641–654. doi:10.1039/C6PY01795A.
2. Puglia, D.; Kenny, J. Chapter 7 - Structure-property relationships of thermoset nanocomposites. In *Thermosets (Second Edition)*, Second Edition ed.; Guo, Q., Ed.; Elsevier: Amsterdam, the Netherlands, 2018; pp. 231–276. doi:10.1016/B978-0-08-101021-1.00007-1.
3. Müller, K.; Bugnicourt, E.; Latorre, M.; Jorda, M.; Echegoyen Sanz, Y.; Lagaron, J.M.; Miesbauer, O.; Bianchin, A.; Hankin, S.; Bölz, U.; Pérez, G.; Jesdinszki, M.; Lindner, M.; Scheuerer, Z.; Castelló, S.; Schmid, M. Review on the Processing and Properties of Polymer Nanocomposites and Nanocoatings and Their Applications in the Packaging, Automotive and Solar Energy Fields. *Nanomaterials* **2017**, *7*, 74–120. doi:10.3390/nano7040074.
4. Duncan, T.V. Applications of nanotechnology in food packaging and food safety: Barrier materials, antimicrobials and sensors. *J. Colloid Interface Sci.* **2011**, *363*, 1–24. doi:10.1016/j.jcis.2011.07.017.
5. Hernández-Muñoz, P.; Cerisuelo, J.P.; Domínguez, I.; López-Carballo, G.; Catalá, R.; Gavara, R. Chapter 8 - Nanotechnology in Food Packaging. In *Nanomaterials for Food Applications*; López Rubio, A.; Fabra Rovira, M.J.; martínez Sanz, M.; Gómez-Mascaraque, L.G., Eds.; Micro and Nano Technologies, Elsevier: Amsterdam, the Netherlands, 2019; pp. 205–232. doi:10.1016/B978-0-12-814130-4.00008-7.
6. Videira-Quintela, D.; Martin, O.; Montalvo, G. Recent advances in polymer-metallic composites for food packaging applications. *Trends Food Sci. Technol.* **2021**, *109*, 230–244. doi:10.1016/j.tifs.2021.01.020.
7. Díez-Pascual, A.M.; Luceño Sánchez, J.A.; Peña Capilla, R.; García Díaz, P. Recent Developments in Graphene/Polymer Nanocomposites for Application in Polymer Solar Cells. *Polymers* **2018**, *10*, 217–238. doi:10.3390/polym10020217.
8. Kuznetsov, I.E.; Akkuratov, A.V.; Troshin, P.A. Chapter 15 - Polymer nanocomposites for solar cells: research trends and perspectives. In *Nanomaterials for Solar Cell Applications*; Thomas, S.; Sakho, E.H.M.; Kalarikkal, N.; Oluwafemi, S.O.; Wu, J., Eds.; Elsevier, 2019; pp. 557–600. doi:10.1016/B978-0-12-813337-8.00015-1.
9. Kumar, S.K.; Ganesan, V.; Riggleman, R.A. Perspective: Outstanding theoretical questions in polymer-nanoparticle hybrids. *J. Chem. Phys.* **2017**, *147*, 020901. doi:10.1063/1.4990501.
10. Starr, F.W.; Douglas, J.F.; Glotzer, S.C. Origin of particle clustering in a simulated polymer nanocomposite and its impact on rheology. *J. Chem. Phys.* **2003**, *119*, 1777–1788. doi:10.1063/1.1580099.
11. Meng, D.; Kumar, S.K.; Cheng, S.; Grest, G.S. Simulating the miscibility of nanoparticles and polymer melts. *Soft Matter* **2013**, *9*, 5417–5427. doi:10.1039/C3SM50460C.
12. Zhao, D.; Di Nicola, M.; Khani, M.M.; Jestin, J.; Benicewicz, B.C.; Kumar, S.K. Role of block copolymer adsorption versus bimodal grafting on nanoparticle self-assembly in polymer nanocomposites. *Soft Matter* **2016**, *12*, 7241–7247. doi:10.1039/C6SM01396A.
13. Koh, C.; Grest, G.S.; Kumar, S.K. Assembly of Polymer-Grafted Nanoparticles in Polymer Matrices. *ACS Nano* **2020**, *14*, 13491–13499. doi:10.1021/acsnano.0c05495.
14. Bieligmeyer, M.; Taheri, S.M.; German, I.; Boisson, C.; Probst, C.; Milius, W.; Altstädt, V.; Breu, J.; Schmidt, H.W.; D'Agosto, F.; Förster, S. Completely Miscible Polyethylene Nanocomposites. *J. Am. Chem. Soc.* **2012**, *134*, 18157–18160. doi:10.1021/ja307297c.
15. Frischknecht, A.L.; Hore, M.J.A.; Ford, J.; Composto, R.J. Dispersion of Polymer-Grafted Nanorods in Homopolymer Films: Theory and Experiment. *Macromolecules* **2013**, *46*, 2856–2869. doi:10.1021/ma302461h.
16. Cobo Sánchez, C.; Wåhlander, M.; Taylor, N.; Fogelström, L.; Malmström, E. Novel Nanocomposites of Poly(lauryl methacrylate)-Grafted Al2O3 Nanoparticles in LDPE. *ACS Appl. Mater. Interfaces* **2015**, *7*, 25669–25678. doi:10.1021/acsami.5b06427.
17. Zhao, W.; Su, Y.; Wen, X.; Wang, D. Manipulating crystallization behavior of poly(ethylene oxide) by functionalized nanoparticle inclusion. *Polymer* **2019**, *165*, 28–38. doi:10.1016/j.polymer.2019.01.019.
18. Zhu, T.; Rahman, M.A.; Benicewicz, B.C. Synthesis of Well-Defined Polyolefin Grafted SiO2 Nanoparticles with Molecular Weight and Graft Density Control. *ACS Macro Lett.* **2020**, *9*, 1255–1260. doi:10.1021/acsmacrolett.0c00398.
19. Matsen, M.W. The standard Gaussian model for block copolymer melts. *Journal of Physics: Condensed Matter* **2001**, *14*, R21–R47. doi:10.1088/0953-8984/14/2/201.
20. Fredrickson, G.H.; Ganesan, V.; Drolet, F. Field-Theoretic Computer Simulation Methods for Polymers and Complex Fluids. *Macromolecules* **2002**, *35*, 16–39. doi:10.1021/ma011515t.
21. García Daza, F.A.; Colville, A.J.; Mackie, A.D. Mean-Field Coarse-Grained Model for Poly(ethylene oxide)-Poly(propylene oxide)-Poly(ethylene oxide) Triblock Copolymer Systems. *Langmuir* **2015**, *31*, 3596–3604. doi:10.1021/la504884m.
22. Shou, Z.; Buxton, G.A.; Balazs, A.C. Predicting the self-assembled morphology and mechanical properties of mixtures of diblocks and rod-like nanoparticles. *Compos. Interfaces* **2003**, *10*, 343–368. doi:10.1163/156855403771953632.
23. Hur, K.; Hennig, R.G.; Escobedo, F.A.; Wiesner, U. Mesoscopic structure prediction of nanoparticle assembly and coassembly: Theoretical foundation. *J. Chem. Phys.* **2010**, *133*, 194108. doi:10.1063/1.3502680.
24. Berezkin, A.V.; Kudryavtsev, Y.V.; Gorkunov, M.V.; Osipov, M.A. Ordering of anisotropic nanoparticles in diblock copolymer lamellae: Simulations with dissipative particle dynamics and a molecular theory. *J. Chem. Phys.* **2017**, *146*, 144902. doi:10.1063/1.4979897.
25. Sides, S.W.; Kim, B.J.; Kramer, E.J.; Fredrickson, G.H. Hybrid Particle-Field Simulations of Polymer Nanocomposites. *Phys. Rev. Lett.* **2006**, *96*, 250601. doi:10.1103/PhysRevLett.96.250601.

26. Kim, J.U.; Matsen, M.W. Positioning Janus Nanoparticles in Block Copolymer Scaffolds. *Phys. Rev. Lett.* **2009**, *102*, 078303. doi:10.1103/PhysRevLett.102.078303.
27. Chao, H.; Hagberg, B.A.; Riggleman, R.A. The distribution of homogeneously grafted nanoparticles in polymer thin films and blends. *Soft Matter* **2014**, *10*, 8083–8094. doi:10.1039/C4SM01188K.
28. Beaudoin, E.; Abecassis, B.; Constantin, D.; Degrouard, J.; Davidson, P. Strain-controlled fluorescence polarization in a CdSe nanoplatelet–block copolymer composite. *Chem. Commun.* **2015**, *51*, 4051–4054. doi:10.1039/C4CC07617F.
29. Beaudoin, E.; Davidson, P.; Abecassis, B.; Bizien, T.; Constantin, D. Reversible strain alignment and reshuffling of nanoplatelet stacks confined in a lamellar block copolymer matrix. *Nanoscale* **2017**, *9*, 17371–17377. doi:10.1039/C7NR05723G.
30. Alexandre, M.; Dubois, P. Polymer-layered silicate nanocomposites: preparation, properties and uses of a new class of materials. *Mater. Sci. Eng. R Rep.* **2000**, *28*, 1–63. doi:10.1016/S0927-796X(00)00012-7.
31. Chang, K.C.; Chen, S.T.; Lin, H.F.; Lin, C.Y.; Huang, H.H.; Yeh, J.M.; Yu, Y.H. Effect of clay on the corrosion protection efficiency of PMMA/Na+-MMT clay nanocomposite coatings evaluated by electrochemical measurements. *Eur. Polym. J.* **2008**, *44*, 13–23. doi:10.1016/j.eurpolymj.2007.10.011.
32. Krook, N.M.; Ford, J.; Maréchal, M.; Rannou, P.; Meth, J.S.; Murray, C.B.; Composto, R.J. Alignment of Nanoplates in Lamellar Diblock Copolymer Domains and the Effect of Particle Volume Fraction on Phase Behavior. *ACS Macro Lett.* **2018**, *7*, 1400–1407. doi:10.1021/acsmacrolett.8b00665.
33. Merritt, S.M.J.; Wemyss, A.M.; Farris, S.; Patole, S.; Patias, G.; Haddleton, D.M.; Shollock, B.; Wan, C. Gas Barrier Polymer Nanocomposite Films Prepared by Graphene Oxide Encapsulated Polystyrene Microparticles. *ACS Appl. Polym. Mater.* **2020**, *2*, 725–731. doi:10.1021/acsapm.9b01041.
34. Hore, M.J.A.; Composto, R.J. Functional Polymer Nanocomposites Enhanced by Nanorods. *Macromolecules* **2014**, *47*, 875–887. doi:10.1021/ma402179w.
35. White, S.I.; DiDonna, B.A.; Mu, M.; Lubensky, T.C.; Winey, K.I. Simulations and electrical conductivity of percolated networks of finite rods with various degrees of axial alignment. *Phys. Rev. B* **2009**, *79*, 024301. doi:10.1103/PhysRevB.79.024301.
36. Ma, X.; Zhu, X.; You, F.; Feng, J.; Wang, M.C.; Zhao, X. Preparation and optical polarization of Ag/epoxy composite films with aligned Ag nanowires. *J. Alloys Compd.* **2014**, *592*, 57–62. doi:10.1016/j.jallcom.2014.01.004.
37. Duan, S.k.; Niu, Q.l.; Wei, J.f.; He, J.b.; Yin, Y.a.; Zhang, Y. Water-bath assisted convective assembly of aligned silver nanowire films for transparent electrodes. *Phys. Chem. Chem. Phys.* **2015**, *17*, 8106–8112. doi:10.1039/C4CP05989A.
38. Meng, L.; Bian, R.; Guo, C.; Xu, B.; Liu, H.; Jiang, L. Aligning Ag Nanowires by a Facile Bioinspired Directional Liquid Transfer: Toward Anisotropic Flexible Conductive Electrodes. *Adv. Mater.* **2018**, *30*, 1706938. doi:10.1002/adma.201706938.
39. Xu, Y.; Ge, D.; Calderon-Ortiz, G.A.; Exarhos, A.L.; Bretz, C.; Alsayed, A.; Kurz, D.; Kikkawa, J.M.; Dreyfus, R.; Yang, S.; Yodh, A.G. Highly conductive and transparent coatings from flow-aligned silver nanowires with large electrical and optical anisotropy. *Nanoscale* **2020**, *12*, 6438–6448. doi:10.1039/C9NR09598E.
40. Yan, L.T.; Popp, N.; Ghosh, S.K.; Böker, A. Self-Assembly of Janus Nanoparticles in Diblock Copolymers. *ACS Nano* **2010**, *4*, 913–920. doi:10.1021/nn901739v.
41. Thorkelsson, K.; Nelson, J.H.; Alivisatos, A.P.; Xu, T. End-to-End Alignment of Nanorods in Thin Films. *Nano Lett.* **2013**, *13*, 4908–4913. doi:10.1021/nl402862b.
42. Liu, Z.; Guo, R.; Xu, G.; Huang, Z.; Yan, L.T. Entropy-Mediated Mechanical Response of the Interfacial Nanoparticle Patterning. *Nano Lett.* **2014**, *14*, 6910–6916. doi:10.1021/nl5029396.
43. Zhu, G.; Huang, Z.; Xu, Z.; Yan, L.T. Tailoring Interfacial Nanoparticle Organization through Entropy. *Acc. Chem. Res.* **2018**, *51*, 900–909. doi:10.1021/acs.accounts.8b00001.
44. Diaz, J.; Pinna, M.; Zvelindovsky, A.; Pagonabarraga, I. Co-assembly of Janus nanoparticles in block copolymer systems. *Soft Matter* **2019**, *15*, 6400–6410. doi:10.1039/C9SM01062A.
45. Diaz, J.; Pinna, M.; Zvelindovsky, A.V.; Pagonabarraga, I. Nonspherical Nanoparticles in Block Copolymer Composites: Nanosquares, Nanorods, and Diamonds. *Macromolecules* **2019**, *52*, 8285–8294. doi:10.1021/acs.macromol.9b01754.
46. Rasin, B.; Lindsay, B.J.; Ye, X.; Meth, J.S.; Murray, C.B.; Riggleman, R.A.; Composto, R.J. Nanorod position and orientation in vertical cylinder block copolymer films. *Soft Matter* **2020**, *16*, 3005–3014. doi:10.1039/D0SM00043D.
47. Sarkar, B.; Alexandridis, P. Block copolymer–nanoparticle composites: Structure, functional properties, and processing. *Prog. Polym. Sci.* **2015**, *40*, 33–62. doi:10.1016/j.progpolymsci.2014.10.009.
48. Thorkelsson, K.; Mastroianni, A.J.; Ercius, P.; Xu, T. Direct Nanorod Assembly Using Block Copolymer-Based Supramolecules. *Nano Lett.* **2012**, *12*, 498–504. doi:10.1021/nl2040089.
49. Krook, N.M.; Tabedzki, C.; Elbert, K.C.; Yager, K.G.; Murray, C.B.; Riggleman, R.A.; Composto, R.J. Experiments and Simulations Probing Local Domain Bulge and String Assembly of Aligned Nanoplates in a Lamellar Diblock Copolymer. *Macromolecules* **2019**, *52*, 8989–8999. doi:10.1021/acs.macromol.9b01324.
50. Rasin, B.; Chao, H.; Jiang, G.; Wang, D.; Riggleman, R.A.; Composto, R.J. Dispersion and alignment of nanorods in cylindrical block copolymer thin films. *Soft Matter* **2016**, *12*, 2177–2185. doi:10.1039/C5SM02442K.
51. Hsu, S.W.; Xu, T. Tailoring Co-assembly of Nanodiscs and Block Copolymer-Based Supramolecules by Manipulating Interparticle Interactions. *Macromolecules* **2019**, *52*, 2833–2842. doi:10.1021/acs.macromol.9b00069.

52. Dong, B.; Huang, Z.; Chen, H.; Yan, L.T. Chain-Stiffness-Induced Entropy Effects Mediate Interfacial Assembly of Janus Nanoparticles in Block Copolymers: From Interfacial Nanostructures to Optical Responses. *Macromolecules* **2015**, *48*, 5385–5393. doi:10.1021/acs.macromol.5b01290.
53. Chen, P.; Yang, Y.; Dong, B.; Huang, Z.; Zhu, G.; Cao, Y.; Yan, L.T. Polymerization-Induced Interfacial Self-Assembly of Janus Nanoparticles in Block Copolymers: Reaction-Mediated Entropy Effects, Diffusion Dynamics, and Tailorable Micromechanical Behaviors. *Macromolecules* **2017**, *50*, 2078–2091. doi:10.1021/acs.macromol.7b00012.
54. Osipov, M.; Ushakova, A. Orientational ordering and spatial distribution of Janus nanoparticles in lamellae diblock copolymers. *J. Mol. Liq.* **2018**, *267*, 330–336. Special Issue Dedicated to the Memory of Professor Y. Reznikov, doi:10.1016/j.molliq.2018.01.048.
55. Patti, A.; Siperstein, F.R.; Mackie, A.D. Phase Behavior of Model Surfactants in the Presence of Hybrid Particles. *J. Phys. Chem. C* **2007**, *111*, 16035–16044. doi:10.1021/jp074486i.
56. Patti, A.; Mackie, A.D.; Siperstein, F.R. Monte Carlo Simulation of Self-Assembled Ordered Hybrid Materials. *Langmuir* **2007**, *23*, 6771–6780. doi:10.1021/la063296g.
57. Patti, A.; Mackie, A.D.; Zelenak, V.; Siperstein, F.R. One-pot synthesis of amino functionalized mesoporous silica materials: using simulations to understand transitions between different structures. *J. Mater. Chem.* **2009**, *19*, 724–732. doi:10.1039/B813016G.
58. Patti, A.; Mackie, A.D.; Siperstein, F.R. Monte Carlo simulations of self-assembling hexagonal and cage-like bifunctional periodic mesoporous materials. *J. Mater. Chem.* **2009**, *19*, 7848–7855. doi:10.1039/B914537K.
59. Patti, A. Monte Carlo simulations of self-assembling star-block copolymers in dilute solutions. *Colloids Surf, A Physicochem. Eng. Asp.* **2010**, *361*, 81–89. doi:https://doi.org/10.1016/j.colsurfa.2010.03.022.
60. Zeleňák, V.; Skřínska, M.; Siperstein, F.R.; Patti, A. Phase evolution during one-pot synthesis of amine modified mesoporous silica materials: Preparation, properties, carbon dioxide adsorption. *Appl. Surf. Sci.* **2019**, *476*, 886–896. doi:https://doi.org/10.1016/j.apsusc.2019.01.146.
61. Campos-Villalobos, G.; Siperstein, F.R.; Charles, A.; Patti, A. Solvent-induced morphological transitions in methacrylate-based block-copolymer aggregates. *J. Colloid Interface Sci.* **2020**, *572*, 133–140. doi:https://doi.org/10.1016/j.jcis.2020.03.067.
62. Patti, A. Molecular Dynamics of Spherical Nanoparticles in Dense Polymer Melts. *J. Phys. Chem. B* **2014**, *118*, 3731–3742. doi:10.1021/jp412440g.
63. Burgos-Mármol, J.J.; Álvarez-Machancoses, Ó.; Patti, A. Modeling the Effect of Polymer Chain Stiffness on the Behavior of Polymer Nanocomposites. *J. Phys. Chem. B* **2017**, *121*, 6245–6256. doi:10.1021/acs.jpcb.7b02502.
64. Burgos-Mármol, J.J.; Patti, A. Unveiling the impact of nanoparticle size dispersity on the behavior of polymer nanocomposites. *Polymer* **2017**, *113*, 92–104. doi:10.1016/j.polymer.2017.01.081.
65. Burgos-Mármol, J.J.; Solans, C.; Patti, A. Effective short-range Coulomb correction to model the aggregation behavior of ionic surfactants. *J. Chem. Phys.* **2016**, *144*, 234904. doi:10.1063/1.4954063.
66. Larson, R.G.; Scriven, L.E.; Davis, H.T. Monte Carlo simulation of model amphiphile-oil–water systems. *J. Chem. Phys.* **1985**, *83*, 2411–2420. doi:10.1063/1.449286.
67. Wang, Y.; Gkeka, P.; Fuchs, J.E.; Liedl, K.R.; Cournia, Z. DPPC-cholesterol phase diagram using coarse-grained Molecular Dynamics simulations. *Biochim. Biophys. Acta Biomembr.* **2016**, *1858*, 2846–2857. doi:https://doi.org/10.1016/j.bbamem.2016.08.005.
68. Kmiecik, S.; Gront, D.; Kolinski, M.; Wieteska, L.; Dawid, A.E.; Kolinski, A. Coarse-Grained Protein Models and Their Applications. *Chem. Rev.* **2016**, *116*, 7898–7936. doi:10.1021/acs.chemrev.6b00163.
69. Salerno, K.M.; Agrawal, A.; Perahia, D.; Grest, G.S. Resolving Dynamic Properties of Polymers through Coarse-Grained Computational Studies. *Phys. Rev. Lett.* **2016**, *116*, 058302. doi:10.1103/PhysRevLett.116.058302.
70. Smith, G.D.; Bedrov, D.; Li, L.; Byutner, O. A molecular dynamics simulation study of the viscoelastic properties of polymer nanocomposites. *J. Chem. Phys.* **2002**, *117*, 9478–9489. doi:10.1063/1.1516589.
71. Wang, M.; Galpaya, D.; Lai, Z.B.; Xu, Y.; Yan, C. Surface functionalization on the thermal conductivity of graphene–polymer nanocomposites. *Int. J. Smart. Nano Mater.* **2014**, *5*, 123–132. doi:10.1080/19475411.2014.904828.
72. Liu, J.; Cao, D.; Zhang, L. Molecular Dynamics Study on Nanoparticle Diffusion in Polymer Melts: A Test of the Stokes-Einstein Law. *J. Phys. Chem. C* **2008**, *112*, 6653–6661. doi:10.1021/jp800474t.
73. Hagita, K.; Morita, H.; Doi, M.; Takano, H. Coarse-Grained Molecular Dynamics Simulation of Filled Polymer Nanocomposites under Uniaxial Elongation. *Macromolecules* **2016**, *49*, 1972–1983. doi:10.1021/acs.macromol.5b02799.
74. Song, J.; Hsu, D.D.; Shull, K.R.; Phelan, F.R.; Douglas, J.F.; Xia, W.; Keten, S. Energy Renormalization Method for the Coarse-Graining of Polymer Viscoelasticity. *Macromolecules* **2018**, *51*, 3818–3827. doi:10.1021/acs.macromol.7b02560.
75. Henry, M.M.; Thomas, S.; Alberts, M.; Estridge, C.E.; Farmer, B.; McNair, O.; Jankowski, E. General-Purpose Coarse-Grained Toughened Thermoset Model for 44DDS/DGEBA/PES. *Polymers* **2020**, *12*. doi:10.3390/polym12112547.
76. Kremer, K.; Grest, G.S. Dynamics of entangled linear polymer melts: A molecular-dynamics simulation. *J. Chem. Phys.* **1990**, *92*, 5057–5086. doi:10.1063/1.458541.
77. Smith, J.S.; Bedrov, D.; Smith, G.D. A molecular dynamics simulation study of nanoparticle interactions in a model polymer-nanoparticle composite. *Compos. Sci. Technol.* **2003**, *63*, 1599 – 1605. doi:10.1016/S0266-3538(03)00061-7.
78. Molinari, N.; Sutton, A.P.; Mostofi, A.A. Mechanisms of reinforcement in polymer nanocomposites. *Phys. Chem. Chem. Phys.* **2018**, *20*, 23085–23094. doi:10.1039/C8CP03281E.
79. Trazkovich, A.J.; Wendt, M.F.; Hall, L.M. Effect of Copolymer Sequence on Local Viscoelastic Properties near a Nanoparticle. *Macromolecules* **2019**, *52*, 513–527. doi:10.1021/acs.macromol.8b02136.

80. Weeks, J.D.; Chandler, D.; Andersen, H.C. Role of Repulsive Forces in Determining the Equilibrium Structure of Simple Liquids. *J. Chem. Phys.* **1971**, *54*, 5237–5247. doi:10.1063/1.1674820.
81. Schultz, A.J.; Hall, C.K.; Genzer, J. Computer simulation of copolymer phase behavior. *J. Chem. Phys.* **2002**, *117*, 10329–10338. doi:10.1063/1.1519839.
82. Schultz, A.J.; Hall, C.K.; Genzer, J. Computer Simulation of Block Copolymer/Nanoparticle Composites. *Macromolecules* **2005**, *38*, 3007–3016. doi:10.1021/ma0496910.
83. He, L.; Zhang, L.; Liang, H. The Effects of Nanoparticles on the Lamellar Phase Separation of Diblock Copolymers. *J. Phys. Chem. B* **2008**, *112*, 4194–4203. doi:10.1021/jp0757412.
84. Plimpton, S. Fast Parallel Algorithms for Short-Range Molecular Dynamics. *J. Comput. Phys.* **1995**, *117*, 1 – 19. doi:10.1006/jcph.1995.1039.
85. Large-scale Atomic/Molecular Massively Parallel Simulator (LAMMPS), stable release 11 August 2017. lammps.sandia.gov (accessed on 24 March 2021).
86. Barkla High Performance Computing Facility. Computing Services Department, University of Liverpool.
87. Shinoda, W.; Shiga, M.; Mikami, M. Rapid estimation of elastic constants by molecular dynamics simulation under constant stress. *Phys. Rev. B* **2004**, *69*, 134103. doi:10.1103/PhysRevB.69.134103.
88. Tuckerman, M.E.; Alejandre, J.; López-Rendón, R.; Jochim, A.L.; Martyna, G.J. A Liouville-operator derived measure-preserving integrator for molecular dynamics simulations in the isothermal–isobaric ensemble. *J. Phys. Math. Gen.* **2006**, *39*, 5629–5651. doi:10.1088/0305-4470/39/19/s18.
89. Gam, S.; Meth, J.S.; Zane, S.G.; Chi, C.; Wood, B.A.; Winey, K.I.; Clarke, N.; Composto, R.J. Polymer diffusion in a polymer nanocomposite: effect of nanoparticle size and polydispersity. *Soft Matter* **2012**, *8*, 6512–6520. doi:10.1039/C2SM25269D.
90. Hales, T.; Adams, M.; Bauer, G.; Dang, T.D.; Harrison, J.; Hoang, L.T.; Kaliszyk, C.; Magron, V.; McLaughlin, S.; Nguyen, T.; et al.. A formal proof of the Kepler Conjecture. *Forum Math. Pi* **2017**, *5*, e2. doi:10.1017/fmp.2017.1.
91. Burgos-Mármol, J.J. Molecular simulation of polymer nanocomposites. PhD thesis, The University of Manchester, Manchester, England, 2017. uk.bl.ethos.719350.
92. Weisstein, E.W. Sphere-Sphere Intersection. From Mathworld – A Wolfram Web Resource. Available online: https://mathworld.wolfram.com/Sphere-SphereIntersection.html (accessed on 24 March 2021).
93. Burgos-Mármol, J.J. LamAnalysis: Software toolkit for Molecular Dynamics of Janus Nanodimers Dispersed in Lamellar Phases of a Block Copolymer. *Zenodo* **2021**. doi:10.5281/zenodo.4660283.
94. Morillo, N.; Patti, A.; Cuetos, A. Brownian dynamics simulations of oblate and prolate colloidal particles in nematic liquid crystals. *J. Chem. Phys.* **2019**, *150*, 204905. doi:10.1063/1.5090975.
95. Hansen, J.P.; McDonald, I.R. *Chapter 11 - Molecular Liquids*, fourth edition ed.; Academic Press: Oxford, 2013; pp. 455 – 510. doi:10.1016/B978-0-12-387032-2.00011-8.
96. Chiu, J.J.; Kim, B.J.; Yi, G.R.; Bang, J.; Kramer, E.J.; Pine, D.J. Distribution of Nanoparticles in Lamellar Domains of Block Copolymers. *Macromolecules* **2007**, *40*, 3361–3365. doi:10.1021/ma061503d.
97. Burgos-Mármol, J.J.; Patti, A. Molecular Dynamics of Janus Nanodimers Dispersed in Lamellar Phases of a Block Copolymer (Dataset 1 of 2). *Zenodo* **2021**. doi:10.5281/zenodo.4665530.
98. Burgos-Mármol, J.J.; Patti, A. Molecular Dynamics of Janus Nanodimers Dispersed in Lamellar Phases of a Block Copolymer (Dataset 2 of 2). *Zenodo* **2021**. doi:10.5281/zenodo.4666523.
